# Linker‐Engineered Dimeric Acceptors Afford Efficient Organic Photocatalytic Hydrogen Evolution via Tailored Nanomorphology for Long‐Lived Charge Accumulation

**DOI:** 10.1002/adma.73648

**Published:** 2026-06-17

**Authors:** Jin‐Woo Lee, Cheng Sun, Yang Song, Guanru Dong, Keren Ai, Stanley Alfred Cazaly, Flurin Eisner, Bumjoon J. Kim, Zeinab Hamid, Iain McCulloch, Yun‐Hi Kim, James R. Durrant

**Affiliations:** ^1^ Department of Chemistry and Centre for Processable Electronics Imperial College London London UK; ^2^ Department of Chemistry Chemistry Research Laboratory University of Oxford Oxford UK; ^3^ Department of Chemical and Biomolecular Engineering Korea Advanced Institute of Science and Technology (KAIST) Daejeon Republic of Korea; ^4^ Qingdao Institute of Bioenergy and Bioprocess Technology Chinese Academy of Sciences Qingdao China; ^5^ Andlinger Center for Energy and the Environment and Department of Electrical and Computer Engineering Princeton University Princeton New Jersey USA; ^6^ School of Engineering and Materials Science Queen Mary University of London London UK; ^7^ Department of Chemistry and RIMA Gyeongsang National University Jinju Republic of Korea

**Keywords:** dimer acceptors, linker engineering, long‐lived charge accumulation, organic photocatalysts, photocatalytic hydrogen evolution

## Abstract

Organic bulk‐heterojunction (BHJ) nanoparticles are promising candidates for solar‐to‐hydrogen conversion. While the development of organic photocatalysts (OPCs) has leveraged advances in organic photovoltaics (OPVs), molecular design rules tailored to photocatalysis remain underdeveloped. Here we introduce linker‐engineered dimeric acceptors that tune self‐assembly and thereby control BHJ nanoparticle morphology, enabling high‐performance OPCs. Two dimer acceptors, DY1 (unfused linker) and DY2 (fused linker), are synthesised from a monomer analogue (MY), establishing a self‐assembly trend of MY > DY2 > DY1. The stronger intermolecular assembly of MY is consistent with a quasi‐core–shell morphology that reduces catalytically accessible donor–acceptor interfaces, whereas the weaker intermolecular assembly of DY1 is associated with a more intermixed morphology and increased recombination losses. In contrast, DY2 exhibits a morphology consistent with improved pathway continuity and sufficient donor/acceptor exposure at the particle surface, supporting enhanced accumulation of long‐lived, surface‐stabilised charges. Consequently, PM6:DY2 OPCs deliver a hydrogen evolution rate of 25.3 µmol h^−1^ cm^−2^, outperforming PM6:MY (1.9 µmol h^−1^ cm^−2^) and PM6:DY1 (11.9 µmol h^−1^ cm^−2^). Notably, this performance trend contrasts with that of the corresponding OPVs, suggesting that photovoltaic design principles do not necessarily translate directly to photocatalysts.

## Graphical abstract

We developed a new series of monomeric (MY) and dimeric acceptors incorporating unfused (DY1) and fused (DY2) linkers, which establish a controlled self‐aggregation trend of MY > DY2 > DY1. The DY2‐based system yields a bulk‐heterojunction nanoparticle morphology that appears to balance phase separation and interfacial accessibility, consistent with efficient long‐lived charge accumulation and superior photocatalytic hydrogen evolution.

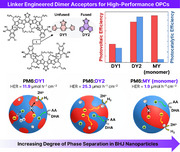

## 1 Introduction

The escalating global energy crisis has created a pressing need for sustainable, carbon‐neutral energy sources. H_2_ stands out as a premier energy carrier due to its high gravimetric energy density and clean combustion profile. Among various production methods, sunlight‐driven photocatalysis offers a direct route to convert solar energy into chemical fuel without the need for an applied electrical bias [1, 2]. In this context, organic photocatalysts (OPCs) based on organic semiconductors have emerged as a promising platform, offering distinct advantages such as high molar extinction coefficients and structurally tunable energy levels that enable broad spectral harvesting from the visible to the near‐infrared (NIR) regions [3, 4, 5, 6, 7, 8, 9, 10]. In particular, donor–acceptor bulk heterojunction (BHJ) nanoparticles (NPs), which maximise exciton dissociation through extensive interfacial areas, have demonstrated significant potential for hydrogen evolution in aqueous media [11, 12, 13].

Despite this promise, the hydrogen evolution rate (HER) of current OPCs remains insufficient for practical deployment. One major bottleneck lies in the severe timescale mismatch between photophysical and chemical processes. In organic semiconductors, exciton decay and charge recombination occur on ultrafast timescales (ps–ns), whereas the proton reduction kinetics at the co‐catalyst surface (e.g., Pt) are orders of magnitude slower (µs–ms) [14, 15, 16]. This disparity leads to substantial charge loss via recombination before electrons can participate in the catalytic reaction. Recent studies indicate that bridging this gap requires the accumulation of long‐lived free charges (polarons) that can persist up to the ms–s timescale in aqueous environments [14, 17]. Such long‐lived populations are widely considered crucial to buffer the kinetic mismatch, allowing sufficient time for electron transfer to slow‐acting catalytic centres.

The capacity to accumulate these long‐lived charges is strongly influenced by the BHJ morphology within the NPs. Optimal charge accumulation demands a delicate structural balance: (1) continuous percolation pathways to transport charges from the interior to the surface, (2) sufficient domain purity to suppress trap‐assisted and bimolecular recombination during transport, and (3) adequate surface exposure of both donor and acceptor domains to facilitate hole scavenging and electron injection to co‐catalysts [18, 19, 20]. Previous reports have highlighted that the NP morphologies, ranging from highly intermixed to core–shell structures, strongly influence H_2_ evolution efficiency [4, 17], yet clear molecular design guidelines to rationally engineer such optimal morphologies are not yet fully established. At the same time, although OPV materials design has provided many of the donor–acceptor pairs now used in OPCs [8, 17, 21, 22, 23], it is unclear to what extent OPV design rules carry over to photocatalytic operation, which demands stable long‐lived charges instead of high photovoltage and efficient charge extraction to electrodes.

The morphological evolution of NPs formed via emulsion solvent evaporation is governed by a complex interplay of thermodynamic and kinetic factors, including surface energy of photocatalysts and surfactants, solubility parameters, and solvent removal rates [24, 25, 26]. Amidst these variables, we posit that the strength of intermolecular interactions within the acceptor phase plays an important role in directing the internal phase separation. In this process, the self‐aggregation tendency of the components competes with the entropy of mixing during solvent removal within the confined nanodroplets [27, 28]. Given that non‐fullerene acceptors typically exhibit stronger aggregation tendencies than donor polymers, modulating these interactions offers an effective strategy to tune the length scale and connectivity of phase‐separated domains [29, 30]. However, a systematic understanding of how acceptor self‐assembly governs the evolution of NP morphology and, consequently, the photophysics and photocatalytic performance is still lacking.

In this study, we propose a molecular design strategy to optimise OPC performance by regulating intermolecular aggregation of acceptor molecules. We synthesised a series of acceptors comprising a monomeric benchmark (**MY**) and two dimeric analogues (**DY1** and **DY2**) with orthogonal linker structures (unfused vs. fused) to systematically modulate steric hindrance and packing behaviour. Notably, MY is structurally analogous to the widely studied Y6 non‐fullerene acceptor [17, 31], but its distinct outer side‐chain architecture results in a markedly higher degree of ordering and crystallinity in NPs, as discussed below. Our results show that dimerisation suppresses the excessive self‐aggregation observed in the monomer, while linker engineering further fine‐tunes the degree of ordering, establishing a gradient of intermolecular interaction strength: **MY** > **DY2** > **DY1**. This variation in interaction strength is reflected in distinct nanomorphologies when blended with the polymer donor PM6. The strongly aggregating **MY** is associated with a quasi‐core–shell morphology that reduces surface‐accessible donor–acceptor interfaces. Conversely, the weaker interaction of **DY1** is associated with a more intermixed morphology that appears to increase recombination losses. By contrast, **DY2**, with intermediate interaction strength, gives rise to a morphology consistent with improved pathway continuity while maintaining sufficient surface exposure for catalysis. Accordingly, PM6:**DY2**‐based OPCs exhibit the most effective long‐lived charge accumulation among the three systems and deliver an HER of 25.3 µmol h^−1^ cm^−2^, together with a mass‐normalised activity of 564 mmol h^−1^ g^−1^. Importantly, the HER trend in OPCs (PM6:**DY2**: 25.3 µmol h^−1^ cm^−2^ > PM6:**DY1**: 11.9 µmol h^−1^ cm^−2^ > PM6:**MY**: 1.9 µmol h^−1^ cm^−2^) contrasts with the power conversion efficiencies (PCEs) of the corresponding OPVs (PM6:**MY**: 17.4% > PM6:**DY2**: 16.4% > PM6:**DY1**: 12.9%), suggesting that the molecular design principles that optimise OPC performance may differ from those governing OPVs. As a result, this work provides a useful materials‐design guideline linking molecular interaction, multi‐scale morphology, and charge kinetics, which can inform the development of high‐performance OPCs.

## 2 Results and Discussion

### 2.1 Materials Design and Optoelectronic Properties

**FIGURE 1:**
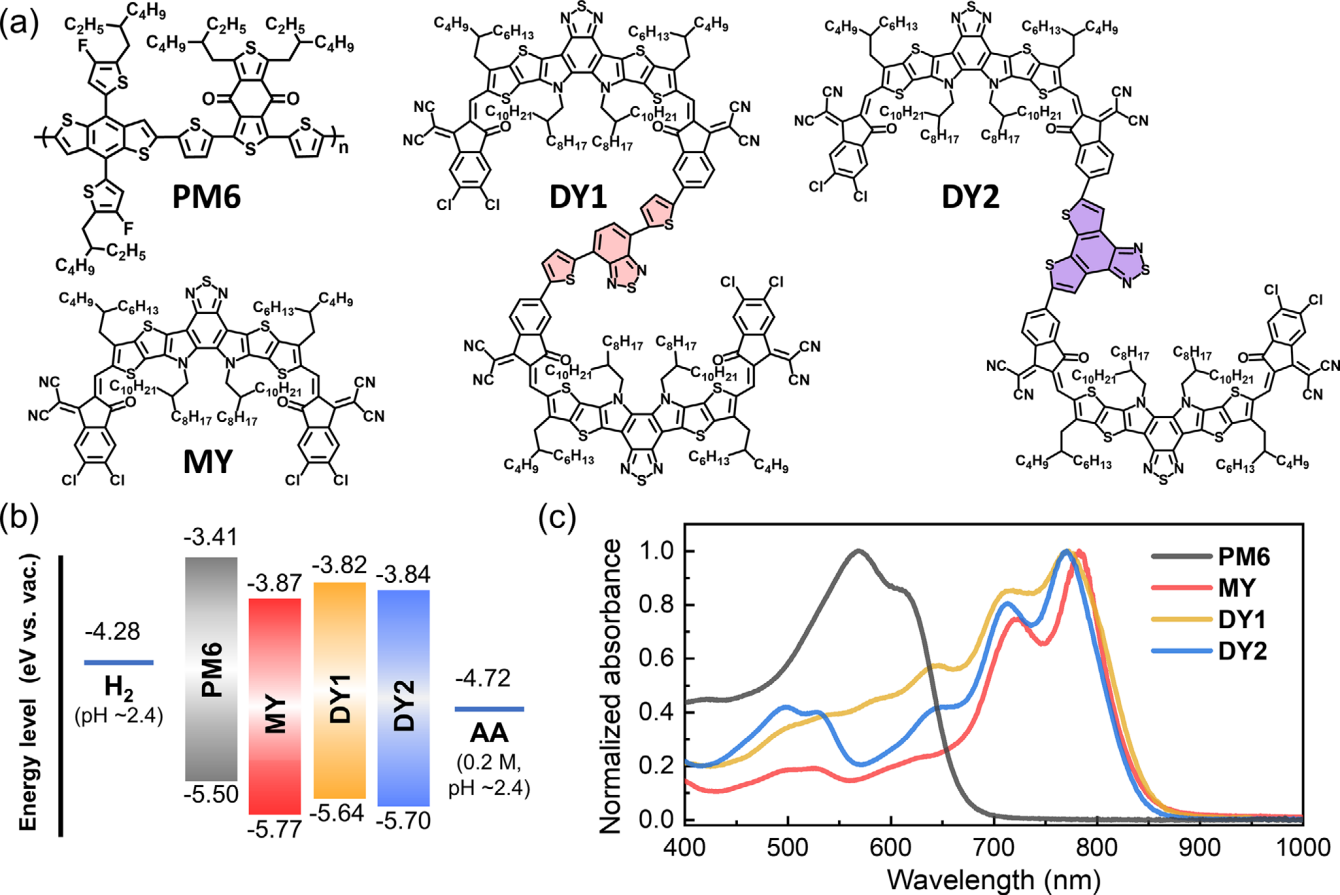
(a) Molecular structures and (b) energy level diagrams (vs. vacuum) of the organic semiconductor materials used in the study. (c) Normalised UV–vis absorption spectra of PM6 and acceptors in NPs.

The chemical structures of the donor and acceptor materials are presented in Figure 1a. **MY** is a high‐performance non‐fullerene acceptor (NFA) structurally analogous to the OPV benchmark L8‐BO [32, 33, 34]. Its ladder‐type backbone enforces a strong intramolecular push–pull character, promoting charge delocalisation and high absorption coefficients [4, 35, 36]. In addition, this design extends absorption into the NIR (∼800–900 nm), broadening the spectral window available for solar‐driven photocatalysis. **MY** further features a rigid conjugated backbone and branched side chains that promote dense packing and strong self‐aggregation [32, 33]. While such ordering is beneficial in OPV thin films, its implications for NP formation are less clear. Unlike spin‐coated films, where morphology is often kinetically frozen by rapid solvent removal, NPs formed via emulsion solvent evaporation experience slower solvent removal in confined droplets, enabling ongoing phase separation and reorganisation [37, 38]. Therefore, OPV‐oriented acceptor design rules may not directly yield the morphologies required for optimal OPC performance.

To investigate how molecular structure influences NP morphology and to establish a controllable handle for tuning acceptor self‐assembly, we designed two dimeric analogues, **DY1** and **DY2**, by linking two **MY**‐based monomer units via an unfused and a fused π‐conjugated linker, respectively (Figure 1a). This dimerization introduces steric hindrance between the two monomeric building blocks, which is expected to suppress excessive self‐aggregation relative to monomeric **MY**. In addition, the linker architecture enables systematic tuning of steric interactions between the building blocks, intermolecular packing, and the electronic properties of the dimer acceptors [32, 39, 40, 41]. The detailed synthetic procedures are described in the Supporting Information, and the successful synthesis of the acceptors was confirmed by nuclear magnetic resonance (NMR) spectroscopy and matrix‐assisted laser desorption/ionization–time of flight (MALDI‐TOF) mass spectrometry (Figures S1–S4).

PM6 was selected as the polymer donor because it provides complementary absorption and is expected to be energetically compatible with these acceptors in OPC NPs [17, 42]. We assessed these optoelectronic considerations using ultraviolet‐visible (UV–vis) absorption spectroscopy and cyclic voltammetry (CV). The acceptor NPs exhibit strong NIR absorption with maxima (*λ*_max_s) at 783, 773, and 771 nm for **MY**, **DY1,** and **DY2**, respectively (Figure 1c). These profiles complement the visible‐range absorption of PM6 NPs (*λ*_max_ = 567 nm), indicating that PM6:acceptor BHJ NPs enable broad photon harvesting across the visible–NIR spectrum (Figure 1c) [43]. CV‐derived frontier molecular orbital (FMO) levels indicate favourable energetic alignment between PM6 and all three acceptors, providing sufficient offsets for interfacial charge transfer (Figure 1b and Figure S5). The acceptor LUMO levels (−3.87 to −3.82 eV) are consistent with the energetic requirements for electron transfer leading to proton reduction at the Pt co‐catalyst (pH ≈ 2.4; −4.28 eV) [12].

To gain insight into how the linker architecture modulates the electronic properties and conformational flexibility of the dimer acceptors, we performed density functional theory (DFT) calculations to obtain optimised geometries (Figure S6). Between the dimer acceptors, **DY1** and **DY2** showed similar dihedral angles across the linker (18°–20°), but **DY1** contains four torsional sites compared with two in **DY2** because of its unfused linker. This higher torsional freedom is expected to increase conformational disorder and impede tight packing [39, 44]. In addition, the DFT‐calculated HOMO and LUMO energy levels of DY2 are lower than those of DY1 (Figure S7), consistent with the trend observed in the CV measurements. This suggests that the fused linker in DY2 provides a more extended and electron‐deficient π‐conjugated framework, giving DY2 a more acceptor‐like electronic character than DY1 [45].

We next examined how these conformational differences translate into optical signatures of intermolecular organisation using UV–vis absorption and photoluminescence (PL) spectroscopy (Figure 1c and Figures S8 and S9). The vibronic intensity ratio (*I*_(0–0)_/*I*_(0–1)_), extracted from the acceptor NP absorption spectra, increased in the order of **DY1** (1.18) < **DY2** (1.27) < **MY** (1.37), consistent with progressively stronger excitonic coupling and more ordered packing [46, 47, 48]. In line with this hierarchy, the maximum absorption coefficient (***α***_max_) of neat acceptor films increased in the order of **DY1** < **DY2** < **MY** (Figure S8 and Table 1). Stokes shifts showed the opposite behaviour, increasing in the order of **MY** < **DY2** < **DY1** (Figure S9 and Table 1), consistent with greater excited‐state relaxation and higher reorganisation energy in the less ordered systems [49, 50]. We further compared the crystallinity and electron‐transport properties of the neat acceptor films (Figures S10 and S11). The relative degree of crystallinity (*r*‐DoC) estimated from the (010) scattering peak in grazing‐incidence wide‐angle X‐ray scattering (GIXS) measurements increased in the order of DY1 < DY2 < MY. Similarly, the electron mobility in film measured by the space‐charge‐limited current (SCLC) method followed the same trend (Table S1) [51]. Together, these optical, structural, and charge‐transport metrics indicate a relative tendency toward ordered acceptor aggregation in the order of **MY** > **DY2** > **DY1**, which is expected to influence phase‐separation kinetics and ultimately the internal morphology of the BHJ NPs.

**TABLE 1:** Optical and electrochemical properties of donor and acceptor materials.

Material	*E*_LUMO_ (eV)^a^	*E*_HOMO_ (eV)^a^	$\lambda_{\max}^{\mathrm{UV}-\mathrm{vis}}$(nm)^b^	$\lambda_{\max}^{\mathrm{PL}}$ (nm)^c^	$\alpha_{\max}^{\mathrm{UV}-\mathrm{vis}}$ (10^5^ × cm^−1^)^d^	*I_(0‐0)_/I_(0‐1)_* ^e^	Stokes shift (nm)^f^
**PM6**	−3.41	−5.50	567	—	—	—	—
**MY**	−3.87	−5.77	783	847	1.21	1.37	64
**DY1**	−3.82	−5.64	773	843	1.03	1.18	70
**DY2**	−3.84	−5.70	771	837	1.12	1.27	66

^a^ Measured by cyclic voltammetry in film.

^b^ Wavelength of maximum UV–vis absorption of NPs.

^c^ Wavelength of maximumPL emission of NPs.

^d^ Maximum absorption coefficient derived from UV–vis absorption profiles in film.

^e^ Intensity ratio between (0−0) and (0−1) peaks in the UV–vis absorption spectra in NPs.

^f^ Estimated from the difference between the absorption and emission maxima in NPs.

### 2.2 Photocatalytic Hydrogen Evolution

**FIGURE 2:**
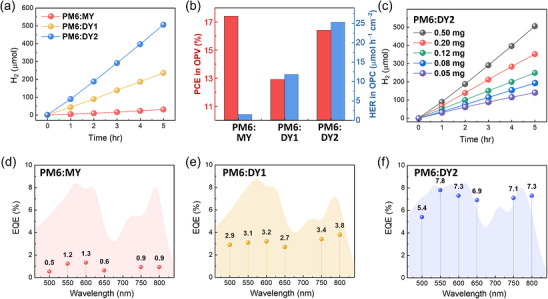
(a) H_2_ evolution over time of the PM6:acceptor‐based OPCs at optimal blend ratio (donor: acceptor = 1:1). (b) Comparison of PCE in OPVs and HER in OPCs among the systems. (c) H_2_ evolution over time of PM6:DY2 (1:1) particles at different photocatalyst amounts in 6 mL aqueous solution. (d–f) EQE spectra of (d) PM6:MY, (e) PM6:DY1, and (f) PM6:DY2‐based OPCs; the profiles in the background are the UV–vis absorption spectra of the corresponding PM6:acceptor NPs measured at the same concentration (0.08 mg mL^−^^1^).

We then investigated the photocatalytic hydrogen evolution activity of the PM6:acceptor BHJ NPs under simulated solar illumination (AM 1.5G, 100 mW cm^−2^). The NPs were fabricated via the mini‐emulsion method using sodium 2‐(3‐thienyl)ethyloxybutylsulfonate (TEBS) as the surfactant, which afforded higher HER activities than the other surfactants tested (Figure S12 and Table S2). Ascorbic acid (0.2 m) was employed as a sacrificial hole scavenger, and Pt (10 wt.%) was loaded as a co‐catalyst via in situ photodeposition [52]. Detailed NP preparation and measurement conditions are provided in the Experimental Section. The time‐dependent H_2_ evolution profiles (at 1:1 blend ratio) and the HERs as a function of donor: acceptor blend ratio are presented in Figure 2a and Figure S13, respectively. All BHJ NPs yielded significantly higher HERs (1.9–25.3 µmol h^−1^ cm^−2^) than the corresponding pristine acceptor NPs (HER = 0.5–1.4 µmol h^−1^ cm^−2^) (Figure S14). This enhancement is attributed to the efficient exciton dissociation facilitated by the donor–acceptor interfaces within the BHJ structure, which promotes superior free charge generation compared to the single‐component NPs [4]. Across all donor: acceptor compositions investigated, each PM6:acceptor system showed its highest activity at a 1:1 blend ratio (Figures S13 and S14). At this composition, PM6:**DY2** achieved a remarkable HER of 25.3 µmol h^−1^ cm^−2^. This value is over an order of magnitude higher than that of PM6:**MY** (HER = 1.9 µmol h^−1^ cm^−2^) and approximately double that of PM6:**DY1** (HER = 11.9 µmol h^−1^ cm^−2^), indicating the decisive impact of acceptor types on photocatalytic activity. We further examined the short‐term operational stability of the PM6:acceptor NPs by performing repeated photocatalytic reactions after refreshing the AA‐containing reaction medium (Figure S15). The PM6:acceptor NPs retained comparable H_2_ evolution activity over repeated cycles, with no substantial decrease in UV–vis absorbance or noticeable change in particle size observed after 5 h of photocatalytic reaction (Figure S16). This suggests that significant photochemical degradation or severe nanoparticle aggregation was not evident within the examined time window.

To determine whether this trend extends to photovoltaic performance, we also fabricated OPV devices using the same PM6:acceptor combinations (blend ratio = 1:1) (Figure S17 and Table S3). The PM6:**MY**, PM6:**DY1,** and PM6:**DY2** devices deliver PCEs of 17.4%, 12.9%, and 16.4%, respectively, largely governed by variations in short‐circuit current density (*J*_sc_) and fill factor (FF) (Figure S17 and Table S3). Interestingly, we observed a divergent structure–property relationship between the two applications: PM6:**MY**, which exhibited the highest PCE in OPVs, showed the poorest catalytic activity in OPCs (Figure 2b). Conversely, PM6:**DY2** outperformed PM6:**MY** by more than 10‐fold in HER despite showing a slightly lower PCE. These results suggest that design rules optimised for solar cells are not necessarily directly transferable to photocatalytic systems, and that dedicated acceptor design guidelines are required for OPCs targeting high hydrogen evolution.

We further investigated the mass‐dependent activity of the best‐performing system, PM6:**DY2** (1:1), by varying the photocatalyst loading from 0.50 mg down to 0.05 mg in 6 mL of aqueous solution (Figure 2c). Interestingly, the HER did not linearly decrease with decreasing concentration, while retaining significant values up to 0.05 mg. Consequently, when the HER is normalised by photocatalyst mass, the PM6:**DY2** system reaches values of 564 mmol h^−1^ g^−1^ (Figure 2c), comparable to representative high‐performing OPC systems reported in the literature (Figure S18 and Table S4). We attribute this high mass activity in part to the large molar extinction coefficients of the organic semiconductors, which enable efficient photon harvesting even in dilute suspensions [13, 21].

Finally, the wavelength‐dependent activity was analysed by measuring the external quantum efficiency (EQE) and the corresponding partial HER contributions across the 500–800 nm range (Figure 2d–f). Consistent with their absorption profiles, all three systems exhibited a broad photoresponse across the measured wavelength range. However, the EQE magnitudes differed significantly depending on the systems. PM6:**MY** exhibits uniformly low EQEs of 0.5–1.3%, whereas PM6:**DY1** reaches intermediate values of 2.7–3.8%. In contrast, PM6:**DY2** displayed the highest EQE values across the entire spectral range of 5.4–7.8%. Notably, this substantial EQE enhancement far exceeds the differences in absorption capability among the acceptors (Figure 2d–f and Figure S8). This suggests that the superior performance of PM6:**DY2** stems from enhanced internal quantum efficiency, driven by more favourable charge generation, transport, and transfer kinetics. The consistently high EQE across the full spectrum of PM6:**DY2** suggests that excitation of both donor‐ and acceptor‐dominated spectral regions can contribute efficiently.

### 2.3 Bulk‐Heterojunction Nanomorphology

To uncover the structural origins of the performance variations, we first examined the particle size distributions using dynamic light scattering (DLS) (Figure S19). All PM6:acceptor NPs (at 1:1 blend ratio) exhibited similar average diameters ranging from 83 to 92 nm, suggesting that differences in particle size are unlikely to be the primary factor governing the distinct HER performance [53]. Consequently, we employed cryogenic transmission electron microscopy (cryo‐TEM) to directly probe their internal microstructures. To assign the donor and acceptor domains, we first established the characteristic lamellar spacings of pristine PM6 and each acceptor. Cryo‐TEM images of neat‐material NPs yielded periodicities of ∼2.2 nm for PM6 and ∼1.6–1.7 nm for the acceptors (Figures S20–S23). These values closely match the *d*‐spacings extracted from the in‐plane (100) peaks in GIXS profiles of the corresponding neat films, with deviations of only ∼0.1–0.2 nm (Figures S24, S25 and Table S5). This cross‐validation supports assigning the fringes observed in cryo‐TEM to the lamellar packing of each material. Notably, although **MY**, **DY1**, and **DY2** are structurally analogous to the widely studied Y6 non‐fullerene acceptor, their NPs appear to exhibit a higher degree of ordering and crystallinity than previously reported Y6 NPs, which showed more amorphous domains in NPs despite exhibiting well‐ordered structures in films [17, 31]. We speculate that this difference arises from the distinct packing behaviour induced by the branched outer side chains of **MY** and its dimeric acceptors [33]. Among these acceptor NPs, **MY** exhibited the highest degree of ordering, consistent with its stronger self‐assembly tendency (Figures S21–S23).

**FIGURE 3:**
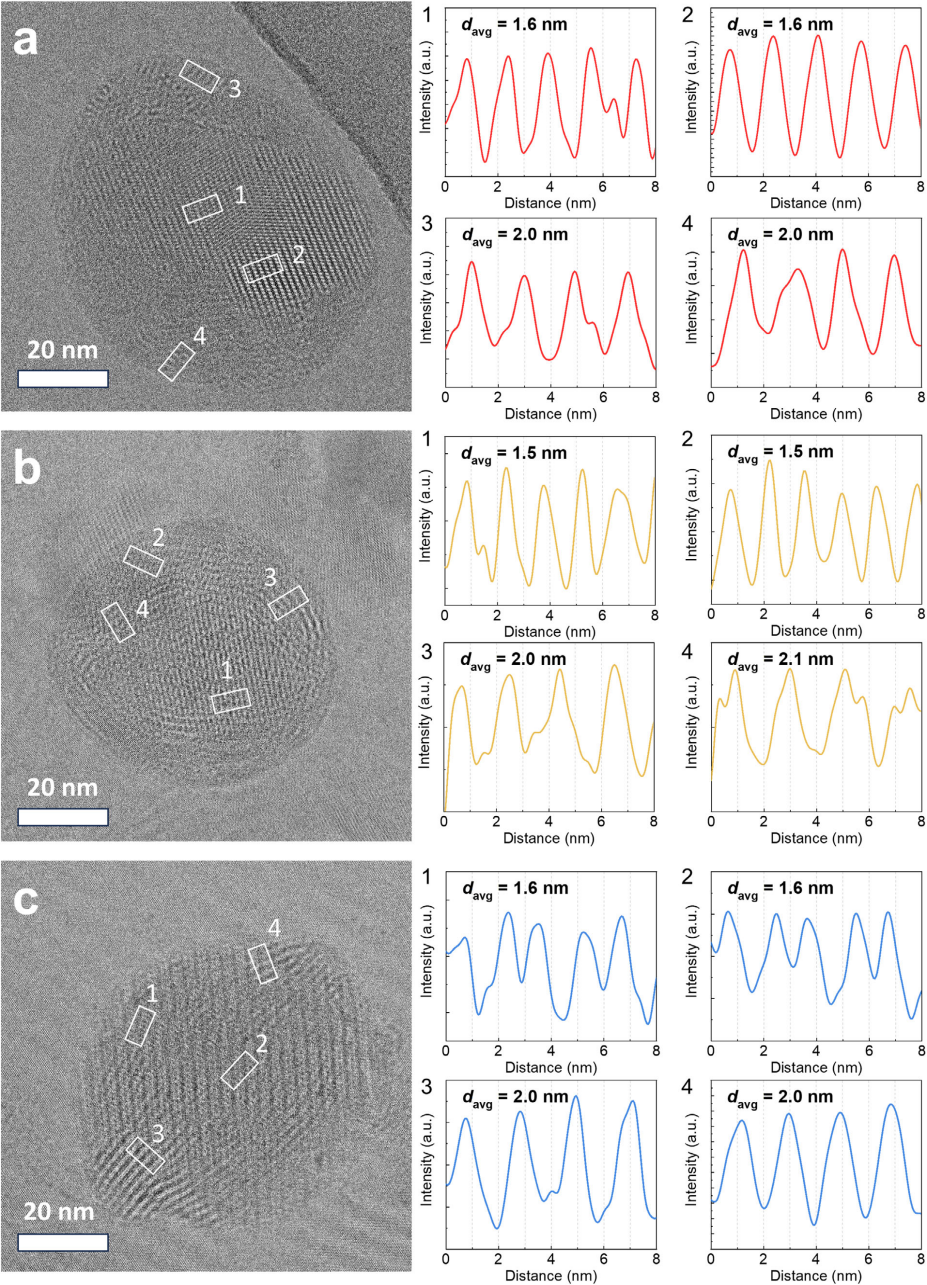
(a–c) Cryo‐TEM images of (a) PM6:MY, (b) PM6:DY1, and (c) PM6:DY2 NPs; the profiles of the periodic spacings highlighted in rectangles are shown on the right.

Figure 3 presents the cryo‐TEM images of the PM6:acceptor BHJ NPs, alongside line‐cut intensity profiles for the highlighted regions. PM6:**MY** NPs exhibited a morphology with a quasi‐core–shell character, featuring an MY‐rich core and a PM6‐enriched shell (Figure 3a). The core regions (regions 1 and 2) displayed regular periodic fringes with a spacing of ∼1.6 nm, matching the lamellar spacing of **MY**, whereas the shell regions (regions 3 and 4) showed periodic spacings of ∼2.0 nm, consistent with the lamellar spacing of PM6. Although traces of the acceptor may remain near the surface, the overall structure suggests that **MY** is preferentially located in the particle interior, leaving the outer shell dominated by the polymer donor. Notably, this core‐shell‐like morphology differs from the intermixed morphology previously reported for Y6‐based BHJ NPs with a similar molecular structure to **MY** [17, 31].

In contrast, the PM6:**DY1** NPs adopted a comparatively intermixed morphology, where domains with periodicities characteristic of **DY1** (regions 1 and 2) and PM6 (regions 3 and 4) are randomly distributed throughout the particle, with no clearly discernible domain boundaries (Figure 3b). This suggests that the unfused dimer **DY1** does not induce strong radial segregation, instead leading to a more blended BHJ morphology than that observed in PM6:**MY**. In comparison, PM6:**DY2** NPs displayed an intermediate morphology that balances phase separation with domain intermixing (Figure 3c). While they retained a recognisable **DY2**‐enriched interior, the outer regions did not form a continuous blocking shell as observed in PM6:**MY**. Instead, the shell appeared to consist of an interpenetrating network where PM6 and **DY2** domains coexist, as evidenced by the observation of lattice fringes corresponding to both components (2.0 nm for PM6 and 1.6 nm for **DY2**) near the particle edges. This structure is consistent with improved pathway continuity extending from the acceptor‐rich core to the surface. Such a morphology is expected to maintain effective charge‐transport channels while allowing sufficient acceptor domains to intersect the particle–water interface, thereby providing active sites for the catalytic reaction.

Inductively coupled plasma mass spectrometry (ICP‐MS) analysis of the NPs after photodeposition of the Pt co‐catalyst further supports this morphology‐based interpretation (Table S6). Since Pt photodeposition occurs through reduction of PtCl_6_^2−^ by photogenerated electrons, Pt may be deposited preferentially at acceptor domains, which function as the main electron‐transporting phase in these BHJ NPs [4, 17]. For the same amount of photocatalyst, the measured Pt concentrations were 22.1, 35.5, and 39.2 ppb for PM6:MY, PM6:DY1, and PM6:DY2, respectively, indicating that Pt deposition in PM6:DY1 and PM6:DY2 was approximately 1.6–1.8 times higher than in PM6:MY (Table S6). This result is consistent with the quasi‐core‐shell character of PM6:MY, where a PM6‐rich shell largely buries the acceptor‐rich core and reduces its accessibility to the particle–water interface. Under these conditions, interfacial electron transfer from acceptor domains to PtCl_6_^2^^−^ is **likely** suppressed [17]. In contrast, the greater surface exposure of acceptor‐rich regions in PM6:DY1 and PM6:DY2 would be expected to promote Pt nucleation and growth [4, 17].

The distinct morphologies observed in the PM6:acceptor NPs might stem from the varying degrees of intermolecular interactions within the acceptor phase, although other factors, such as the surface energies of the photocatalysts and surfactants, solubility parameters, and solvent removal kinetics, may also contribute [24, 25, 26]. The stronger intermolecular assembly of **MY** likely promotes the formation of relatively large and crystalline acceptor domains during blend NP precipitation. In contrast, the weaker intermolecular interactions of **DY1** appear to reduce this driving force, favouring greater mixing with PM6 and resulting in a more intermixed BHJ morphology. **DY2** represents an intermediate scenario; its interaction strength seems sufficient to induce the formation of crystalline domains yet allows for partial mixing with the donor polymer. Consequently, PM6:**DY2** evolves into a balanced morphology featuring a **DY2**‐enriched interior and a percolated PM6/**DY2** network at the surface, as supported by our structural analysis.

### 2.4 Photophysical Properties

**FIGURE 4:**
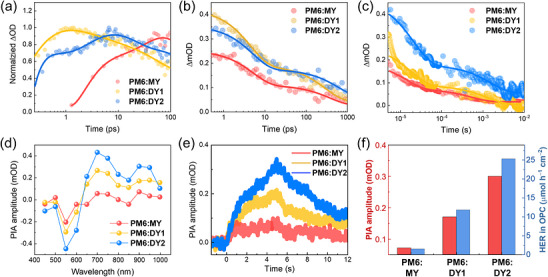
(a) Deconvoluted uf‐TAS kinetics of the PM6 ground‐state bleach under acceptor‐selective excitation at 780 nm (30 µJ cm^−2^), assigned to hole transfer from acceptor excitons to PM6. (b) uf‐TAS kinetics at 1200–1300 nm under the same excitation conditions, assigned to recombination of separated electron–hole pairs in PM6:acceptor NPs. (c) µs–ms TAS kinetics of PM6:acceptor NPs, assigned to recombination of separated charges, recorded under 530 nm excitation (200 µJ cm^−2^) and probed at 950 nm. (d) PIA spectra of PM6:acceptor NPs under 530 nm LED excitation. (e) PIA kinetics of accumulated electron and hole polarons monitored at 900 nm under 530 nm LED excitation. (f) Comparison of PIA amplitudes and HER values for PM6:acceptor OPCs, highlighting the correlation between long‐lived charge accumulation and photocatalytic activity.

To elucidate how the distinct nanomorphologies translate into the observed differences in hydrogen‐evolution performance, we investigated the photophysical response of the PM6:acceptor NPs. In all spectroscopic measurements, the absorbance of the dispersions was adjusted to a comparable value (∼0.6 OD at excitation wavelength) so that the overall light absorption was kept similar across samples under the applied excitation conditions. Ultrafast transient absorption spectroscopy (uf‐TAS) was first employed to probe charge generation and early‐time recombination kinetics. Under acceptor‐selective excitation at 780 nm, we monitored the rise of the PM6 ground‐state bleach (GSB) to extract the kinetics of hole transfer from acceptor excitons to PM6 (Figure 4a). Detailed datasets and global analysis are provided in Figures S26–S28. The hole transfer process in PM6:**MY** is slower than in the other two systems, exhibiting a rise half‐time of ∼5 ps (Figure 4a). In contrast, both PM6:**DY1** and PM6:**DY2** display much faster kinetics, with more than half of the hole‐transfer process completed within ∼1 ps.

These observations are further corroborated by steady‐state PL measurements. When PM6 was predominantly excited, PM6 emission was quenched by more than 98% in all PM6:acceptor blend NPs, suggesting efficient PM6 exciton quenching across the three systems (Figure S29). By contrast, under selective acceptor excitation (λ_ex_ = 780 nm), the dimer‐based NPs exhibited pronounced PL quenching (compared to the pristine acceptor emission) of 60% for PM6:**DY1** and 52% for PM6:**DY2**, which significantly surpass the 40% observed for PM6:**MY** (Figure S30). The time‐resolved photoluminescence (TRPL) measurements further support this trend. The PM6:acceptor blend NPs displayed acceptor‐dependent biexponential decay profiles, with short (τ_1_) and long (τ_2_) components commonly assigned to interfacial exciton quenching/dissociation and excitons in acceptor‐rich domains that must diffuse to the interfaces prior to dissociation, respectively (Figure S31) [54, 55]. While PM6:**MY** exhibited relatively long decay lifetimes (τ_1_ = 0.7 ns and τ_2_ = 4.0 ns), both PM6:**DY1** and PM6:**DY2** displayed accelerated decay dynamics with notably shorter lifetime values (τ_1_ = 0.5–0.6 ns; τ_2_ = 2.7–3.1 ns). Collectively, these spectroscopic findings are consistent with the cryo‐TEM‐derived morphologies. In PM6:**MY**, the pronounced core–shell structure effectively confines MY to the core, forcing excitons to diffuse long distances to the D–A interface, thereby retarding hole transfer. Conversely, the interpenetrating networks in PM6:**DY1** and PM6:**DY2** minimise exciton diffusion limitations, enabling ultrafast charge separation.

We subsequently examined the recombination dynamics of photogenerated charges on the nanosecond timescale (Figure 4b). Specifically, we monitored the transient absorption kinetics at 1200–1300 nm under acceptor‐selective excitation at 780 nm, which probes the photoinduced absorption of polaron species (PM6^+^ and acceptor^−^) (Figure 4b and Figure S27). The initial polaron absorption amplitudes of PM6:**DY1** and PM6:**DY2** were higher than that of PM6:**MY**, consistent with the more efficient charge transfer occurring in the dimer‐based systems (Figure 4b). The subsequent decay traces, however, revealed distinct recombination behaviours. While PM6:**DY1** initially yielded a slightly higher polaron density than PM6:**DY2**, its signal decayed more rapidly, resulting in a larger residual polaron population in PM6:**DY2** beyond 1 ns (Table S7). PM6:**MY** exhibited the weakest initial signal yet a slower recombination rate than PM6:**DY1**. This behaviour reflects inefficient charge generation, coupled with suppressed recombination arising from spatial isolation within the core–shell structure. Consequently, the population of polarons surviving into the nanosecond regime follows the order PM6:**MY** < PM6:**DY1** < PM6:**DY2**, mirroring the trend in HER performance. For instance, the polaron absorption intensities at 1 ns for PM6:**MY**, PM6:**DY1**, and PM6:**DY2** were 0.04, 0.07, and 0.10 mOD, respectively (Figure 4b). Since the pristine acceptor NPs exhibited very similar exciton decay kinetics, with half‐decay times of ∼10 ps (Figures S32 and S33), the distinct polaron kinetics of the PM6:acceptor blend NPs are more reasonably attributed to differences in charge recombination dynamics. A similar tendency was also observed in the uf‐TAS measurements under predominant PM6 excitation at 580 nm; although PM6:DY1 exhibited the fastest PM6 exciton decay, PM6:DY2 retained more persistent polaron signals after charge generation (Figures S34–S36). We extended this investigation to longer timescales (µs–ms) using TAS measurements (Figure S37 and Table S8). The decay profiles recorded at 950 nm (under 530 nm excitation) probe the long‐lived population of PM6 and acceptor polarons (Figure 4c and Figure S37). Consistent with the nanosecond‐regime results, the initial polaron signals in the microsecond regime increased in the order PM6:**MY** < PM6:**DY1** < PM6:**DY2**. Notably, PM6:**DY2** retained a substantially larger polaron population than the other two systems even beyond 100 µs, indicating the most efficient accumulation of long‐lived charges in the PM6:**DY2** NPs.

To further characterise the fraction of charges persisting beyond the TAS time window, we performed photoinduced absorption (PIA) spectroscopy under continuous LED illumination (Figure 4d,e) [56]. The dispersions were excited for 5 s to allow charge accumulation, after which the LED was switched off to record the subsequent decay kinetics. All samples were purged with Ar to eliminate interference from dissolved oxygen. In this regime, the signal reflects a quasi‐steady‐state population of long‐lived charges capable of accumulating on the timescale of seconds. In organic NP photocatalysts, such slowly decaying PIA responses are typically attributed to charges stabilised at or near the NP surface by the polarization of the surrounding aqueous medium [17, 57]. This surface‐stabilised population is distinct from the rapidly decaying bulk charges probed by pulsed‐laser TAS, which predominantly reflect geminate and non‐geminate recombination within the particle interior. Given that the magnitude of these second‐timescale PIA signals has been shown to track the hydrogen‐evolution rate, the ability of a system to accumulate and sustain these long‐lived surface charges is closely linked to its photocatalytic performance.

Figure 4d,e displays the PIAs spectra after 5 s of 530 nm excitation and the corresponding decay kinetics at 900 nm, respectively. Across all spectral features, including the PM6 ground‐state bleach (500–600 nm), the PM6 polaron absorption (700–800 nm), and the combined polaron feature (>800 nm), the signal intensity consistently increases in the order PM6:**MY** < PM6:**DY1** < PM6:**DY2** (Figure 4d). This trend indicates that the effective population of charges surviving to the second timescale follows the same hierarchy. The decay traces at 900 nm further highlight the superior charge persistence of PM6:**DY2** (Figure 4e). Specifically, PM6:**DY2** accumulated a substantially larger population of long‐lived polarons during the illumination period and retains a much higher residual amplitude than PM6:**MY** and PM6:**DY1** even 7 s after the light is switched off. Plotting the PIA amplitude at 900 nm after 5 s of excitation against the HER activity revealed a monotonic relationship across the three systems, supporting the idea that long‐lived charge accumulation contributes to photocatalytic activity. Considering that proton reduction at the Pt co‐catalyst occurs on timescales much slower than bulk recombination, the exceptional ability of PM6:**DY2** to generate and stabilise these long‐lived charges is likely a central factor driving its enhanced hydrogen‐evolution performance. To further examine whether these accumulated charges are involved in the subsequent catalytic charge‐transfer steps, we measured the PIA responses under AA‐added and AA/Pt‐added conditions (Figures S38 and S39). Addition of AA strongly suppressed the PM6 hole‐polaron signals, indicating efficient hole scavenging by AA. At the same time, the residual long‐lived electron signals still followed the order PM6:MY < PM6:DY1 < PM6:DY2, showing that the superior electron accumulation in PM6:DY2 is maintained under hole‐scavenging conditions. Further addition of Pt substantially reduced the electron‐related PIA signals, consistent with efficient extraction and consumption of accumulated electrons at Pt sites during HER.

**FIGURE 5:**
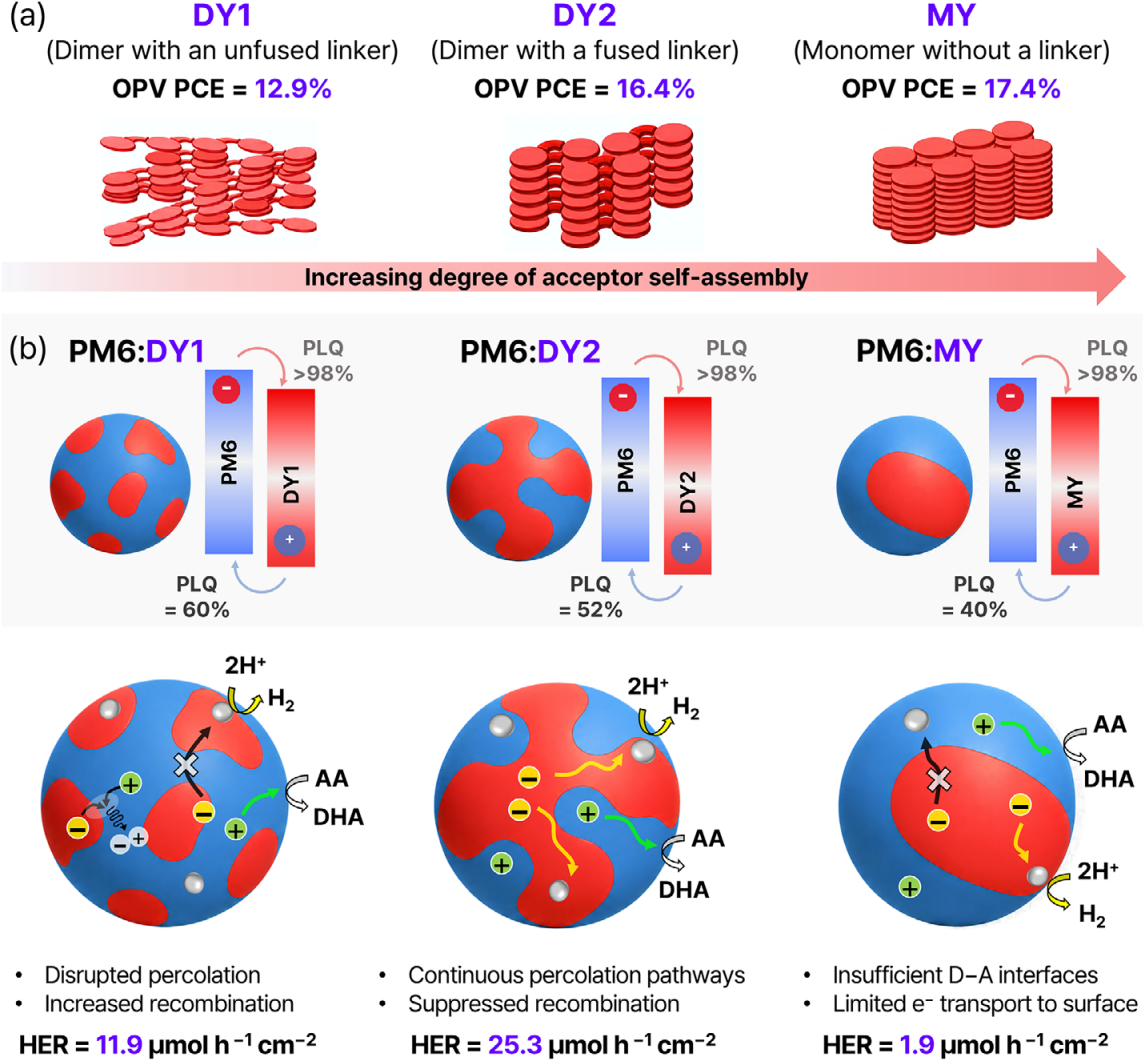
Schematic summary of the relationship between acceptor self‐assembly, BHJ nanoparticle morphology, and photocatalytic H_2_ evolution. (a) Increasing degree of acceptor self‐assembly from DY1 to DY2 to MY. (b) Proposed morphologies, photoluminescence quenching efficiencies, and charge‐transport/photocatalytic pathways of PM6:DY1, PM6:DY2, and PM6:MY BHJ nanoparticles, constructed from the experimental observations.

Based on these comprehensive analyses, schematics illustrating the proposed morphologies and charge kinetics for the three PM6:acceptor NPs are presented in Figure 5. By comparing a monomer acceptor (**MY**) with two dimeric acceptors containing either an unfused linker (**DY1**) or a fused π‐conjugated linker (**DY2**), we examined how the degree of acceptor self‐assembly influences BHJ NP morphology and photocatalytic function. Structural analyses revealed a consistent ordering trend of **DY1** < **DY2** < **MY** in both films and NPs (Figure 5a). In PM6‐based OPVs, this trend correlated with a gradual increase in PCE from PM6:**DY1** (12.9%) to PM6:**DY2** (16.4%) and PM6:**MY** (17.4%), indicating that stronger acceptor ordering can benefit photovoltaic charge transport and extraction. In contrast, the photocatalytic HER activity of the corresponding BHJ NPs followed a different trend, highlighting that photocatalysis requires not only efficient charge generation and transport, but also long‐lived charge accumulation and interfacial electron transfer to surface Pt sites.

PM6:**MY**, with the strongest acceptor self‐assembly, forms a quasi‐core–shell morphology, in which a crystalline **MY**‐rich core is largely covered by a PM6‐enriched shell (Figure 5b). In this configuration, acceptor excitons generated in the interior must diffuse to the D–A interface, which slows hole transfer. Furthermore, while **MY** domains are not completely isolated, their exposure to the aqueous environment is severely restricted by the polymer shell. This limited surface accessibility is expected to hinder interfacial electron transfer to the co‐catalyst, leading to sparse Pt photodeposition and poor stabilisation of surface charges, ultimately resulting in the lowest HER (1.9 µmol h^−1^ cm^−2^).

In contrast, PM6:**DY1**, with the weakest acceptor self‐assembly, forms a relatively intermixed BHJ morphology compared to PM6:**MY**, with donor and acceptor domains randomly distributed throughout the NP (Figure 5b). While this structure provides a large interfacial area for fast exciton dissociation, as evidenced by strong PL quenching and rapid uf‐TAS kinetics, the lack of continuous, well‐defined domains forces charge carriers to traverse compositionally mixed regions. This is expected to increase the probability of non‐geminate recombination during transport. Although the extensive surface exposure of acceptor domains allows for more extensive Pt deposition, the accumulated charges are less effectively stabilised and recombine more readily, resulting in moderate HER performance (11.9 µmol h^−1^ cm^−2^).

PM6:**DY2** exhibits an intermediate morphology that balances the high crystallinity of **MY** with the intermixing tendency of **DY1** (Figure 5b). It features a **DY2**‐enriched interior connected to a mixed PM6/**DY2** shell via percolation pathways. This structure avoids the excessive phase segregation of PM6:**MY** while maintaining better domain connectivity than PM6:**DY1**. Consequently, it preserves sufficient D–A interfaces for exciton dissociation and provides continuous transport channels to the surface. The presence of acceptor domains within the mixed shell can ensure the formation of adequate active sites for electron transfer. This balanced microstructure **is consistent with** the ability of PM6:**DY2** to suppress recombination and stabilise surface charges, delivering the highest hydrogen‐evolution activity (25.3 µmol h^−1^ cm^−2^) among the investigated systems. More importantly, in addition to previous studies that modulated BHJ NP morphology through blend composition, surfactants, or material types and combinations [4, 10, 17, 31], this work shows that acceptor self‐assembly can also play an important role in defining photocatalytically relevant morphology. In this respect, our results provide a useful framework for connecting molecular design with nanoparticle morphology and photocatalytic function in hydrogen‐evolving OPCs.

## 3 Conclusions

In summary, we demonstrated that tuning intermolecular interactions in NFAs provides a molecular handle to programme BHJ NP morphology for photocatalytic hydrogen evolution. Systematic comparison of the monomer **MY** and its dimeric analogues (**DY1** and **DY2**) suggests that differences in acceptor self‐aggregation are closely linked to phase separation and the resulting BHJ NP morphology. The stronger aggregation tendency of **MY** is consistent with a quasi‐core– shell morphology in which surface‐accessible heterojunctions are reduced, likely limiting productive interfacial charge transfer and contributing to the lowest hydrogen‐evolution activity. Conversely, the more sterically disrupted **DY1** is associated with a more intermixed PM6:**DY1** morphology compared to PM6:**MY**, which appears to promote greater recombination losses. By contrast, the fused dimer **DY2** exhibits a morphology consistent with improved pathway continuity while retaining donor/acceptor co‐exposure at the particle surface. This architecture is consistent with more efficient accumulation and stabilisation of long‐lived, surface‐associated charges, which likely contributes to bridging the kinetic mismatch between ultrafast charge generation and slow proton reduction. Consequently, PM6:**DY2** reaches an HER of 25.3 µmol h^−1^ cm^−2^, exceeding those of the corresponding PM6:**MY** (HER = 1.9 µmol h^−1^ cm^−2^) and PM6:**DY1** (HER = 11.9 µmol h^−1^ cm^−2^) systems. The inverted OPV–OPC performance ordering in this material set suggests that OPV‐optimised design does not necessarily translate directly to photocatalysis and highlights surface‐accessible, well‐connected BHJ morphologies as a promising target for OPCs.

## Conflicts of Interest

The authors declare no conflicts of interest.

## Supporting information




**Supporting File**: adma73648‐sup‐0001‐SuppMat.pdf.

## Data Availability

The data that support the findings of this study are available from the corresponding author upon reasonable request.
