# Supplementary material for: Linker‐Engineered Dimeric Acceptors Afford Efficient Organic Photocatalytic Hydrogen Evolution via Tailored Nanomorphology for Long‐Lived Charge Accumulation
Source: Adv Mater. 2026 Jun 17;38(41):e73648. doi: 10.1002/adma.73648 (PMC13393979; doi:10.1002/adma.73648)
Supplement: Supplementary file 1 — Supporting File: adma73648‐sup‐0001‐SuppMat.pdf. [file ADMA-38-e73648-s001.pdf]

Supporting Information for

Linker-Engineered Dimeric Acceptors Afford Efficient Organic Photocatalytic Hydrogen Evolution via Tailored Nanomorphology for Long-Lived Charge Accumulation

Jin-Woo Lee[1,2,3], Cheng Sun[4], Yang Song[5], Guanru Dong[2], Keren Ai[1], Stanley Alfred Cazaly[6], Flurin Eisner[6], Bumjoon J. Kim[3], Zeinab Hamid[2], Iain McCulloch[2,5*], Yun-Hi Kim[7*], and James R. Durrant[1,2*]*

[1]Department of Chemistry and Centre for Processable Electronics, Imperial College London, UK

[2]Department of Chemistry, Chemistry Research Laboratory, University of Oxford, Oxford, UK

[3]Department of Chemical and Biomolecular Engineering, Korea Advanced Institute of Science and Technology (KAIST), Daejeon, Republic of Korea

[4]Qingdao Institute of Bioenergy and Bioprocess Technology, Chinese Academy of Sciences, Qingdao, China

[5]Andlinger Center for Energy and the Environment and Department of Electrical and Computer Engineering, Princeton University, New Jersey, US

[6]School of Engineering and Materials Science, Queen Mary University of London, UK

[7]Department of Chemistry and RIMA, Gyeongsang National University, Jinju, Republic of Korea.

[*]All correspondence should be addressed to J. R. Durrant (E-mail: james.durrant@chem.ox.ac.uk), Y.-H. Kim (E-mail: ykim@gnu.ac.kr), I. McCulloch (E-mail: iain.mcculloch@chem.ox.ac.uk), or J.-W. Lee (E-mail: jinwoo.lee@chem.ox.ac.uk).

Experimental Section

Materials: All solvents and reagents were purchased from Sigma-Aldrich. All solvents were purified prior to use. 2-(3-thienyl)ethyloxybutylsulfonate (TEBS**)** was purchased from Solaris Chem. Poly[(2,6-(4,8-bis(5-(2-ethylhexyl)-4-fluorothiophen-2-yl)-benzo[1,2-b:4,5-b']dithiophene))-alt-(5,5-(1',3'-di-2-thienyl-5',7'-bis(2-ethylhexyl)benzo[1',2'-c:4',5'-c']dithiophene-4,8-dione))] (PM6) was purchased from Solarmer Co. 4,7-bis(5-(trimethylstannyl)thiophen-2-yl)benzo[c][1,2,5]thiadiazole and 5,8-bis(trimethylstannyl)dithieno[3',2':3,4;2'',3'':5,6]benzo[1,2-c][1,2,5]thiadiazole were purchased from Sunatech Co. All the other eluents and materials were purchased from Sigma Aldrich Co. and Tokyo Chemical Industry Co. 3,9-Bis(2-butyloctyl)-12,13-bis(2-octyldodecyl)-12,13-dihydro-[1,2,5]thiadiazolo[3,4-e]thieno[2'',3'':4',5']thieno[2',3':4,5]pyrrolo[3,2-g]thieno[2',3':4,5]thieno[3,2-b]indole-2,10-dicarbaldehyde (Compound 1), 2-(5,6-dichloro-3-oxo-2,3-dihydro-1H-inden-1-ylidene)malononitrile (INCN-2Cl), and Y5-2Cl/Br-In were synthesized following the methods from the previous literature.[1]

(1) Synthesis of MY

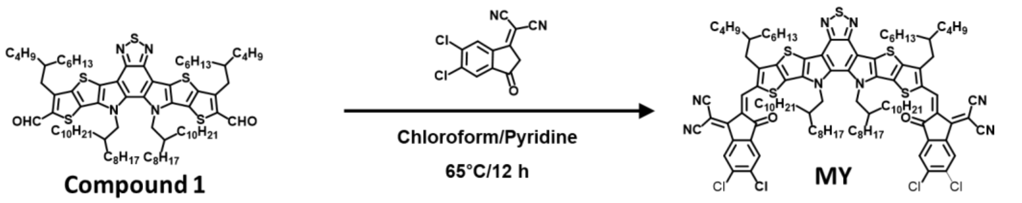

Scheme S1. Synthetic scheme for MY.

INCN-2Cl (470 mg, 1.8 mmol) and Compound 1 (500 mg, 0.36 mmol) were added to a solvent mixture of chloroform (50 mL) and pyridine (2 mL) (**Scheme S1**). The mixture was reacted in an oil bath at 65 °C overnight. After the removal of residual solvents at low pressure (< 300 mbar) using a rotary evaporator, the product was purified by a silica gel column chromatography using hexane/dichloromethane (3:2) as the eluent. The final MY product had a reaction yield of 80% (550 mg).

^{1}H NMR (500 MHz, $CDCl_3$) δ 9.10 (s, 2H), 8.73 (s, 2H), 7.92 (s, 2H), 4.82 (d, *J* = 7.8 Hz, 4H), 3.10 (d, *J* = 7.4 Hz, 4H), 2.22 – 1.94 (m, 4H), 1.43 – 0.98 (m, 96H), 0.90 – 0.75 (m, 24H).

MS (MALDI-TOF) m/z: [M + H]+ calculated for $C_{108}H_{138}Cl_4N_8O_2S_5$, 1881.83, found: 1881.25.

(2) Synthesis of DY1

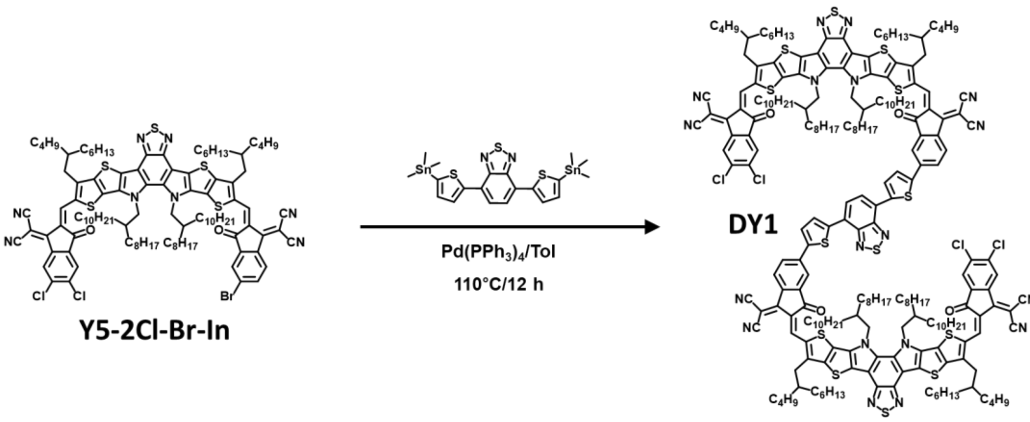

Scheme S2. Synthetic scheme for DY1.

Y5-2Cl/Br-In (380 mg, 0.2 mmol), 4,7-bis(5-(trimethylstannyl)thiophen-2-yl)benzo[c][1,2,5]thiadiazole (51 mg, 0.08 mmol), and $Pd(PPh_3)_4$ (4.6 mg, 0.004 mmol) were combined in a 100 mL two-necked flask. Anhydrous toluene (50 mL) was added under the argon atmosphere (**Scheme S2**). The mixture was reacted for 12 h at 110 °C. After the reaction, residual solvents were removed at low pressure (< 300 mbar), and the product was purified by silica-gel column chromatography using hexane/ chloroform (1:1) as eluent to yield DY1 as black solid (250 mg, 67%).

^{1}H NMR (500 MHz, $CDCl_3$) δ 9.21-8.82 (m, 4H), 8.81-8.56 (m, 4H), 8.25-8.13 (m, 4H), 8.05-7.92 (m, 4H), 7.86-7.67 (m, 4H), 4.92-4.85 (m, 8H), 3.37-2.72 (m, 8H), 2.38-1.95 (m, 8H), 1.47-0.94 (m, 192H), 0.92-0.76 (m, 48H).

MS (MALDI-TOF) m/z: [M + H]+ calculated for $C_{230}H_{284}Cl_4N_{18}O_4S_{13}$, 3921.77, found: 3928.50.

(3) Synthesis of DY2

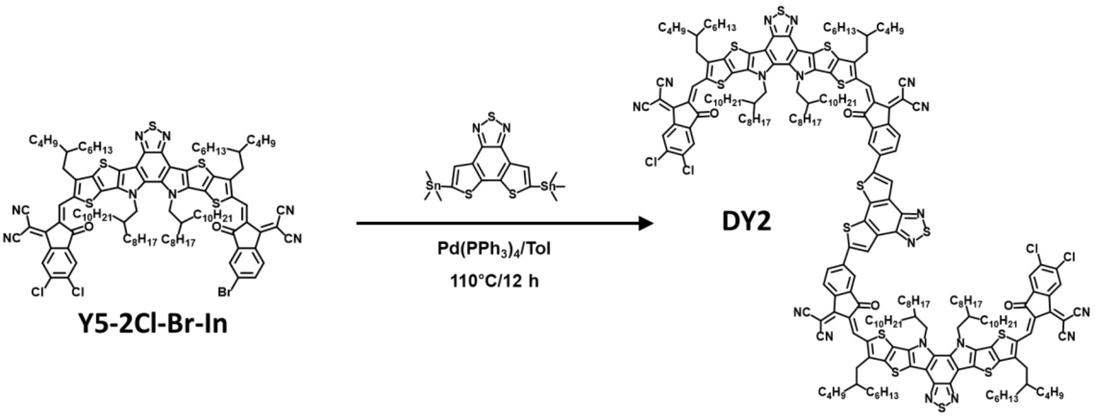

Scheme S3. Synthetic scheme for DY2.

Y5-2Cl/Br-In (380 mg, 0.2 mmol), 5,8-bis(trimethylstannyl)dithieno[3',2':3,4;2",3":5,6]benzo[1,2-c][1,2,5]thiadiazole (46 mg, 0.08 mmol), and $Pd(PPh_3)_4$ (4.6 mg, 0.004 mmol) were combined in a 100 mL two-necked flask (**Scheme S3**). Anhydrous toluene (50 mL) was added under the argon atmosphere. The mixture was reacted for 12 h at 110 °C. After the reaction, residual solvents were removed at low pressure (< 300 mbar), and the product was purified by silica-gel column chromatography using hexane/chloroform (1:2) as eluent to yield DY2 as black solid (230 mg, 62%).

^{1}H NMR (500 MHz, $CDCl_3$) δ 9.16-8.91 (m, 4H), 8.86-8.58 (m, 4H), 8.42-8.22 (m, 2H), 8.17-7.83 (m, 4H), 7.78-7.64 (m, 2H), 5.02-4.85 (m, 8H), 3.35-2.70 (m, 8H), 2.38-2.18 (m, 4H), 2.11-1.95 (m, 4H), 1.45 – 0.95 (m, 192H), 0.90 – 0.75 (m, 48H).

MS (MALDI-TOF) m/z: [M + H]+ calculated for $C_{226}H_{280}Cl_4N_{18}O_4S_{13}$, 3869.74, found: 3873.50.

Characterizations: Bruker Avance Drx 500 MHz NMR spectrometers were used to measure ^{1}H NMR spectra. The chemical shifts in the spectra have units of ppm. Bruker Autoflex MALDI-ToF mass spectrometer was used to measure the molecular weights of the acceptor materials. Cyclic voltammetry (CV) was performed using an EG&G PARC model 273A potentiostat/galvanostat. Measurements were conducted at a scan rate of 50 mV s^{-1} in a nitrogen-degassed anhydrous acetonitrile solution containing 0.1 M tetrabutylammonium perchlorate as the supporting electrolyte. A glassy carbon electrode, a platinum wire, and an Ag/AgCl electrode were used as the working, counter, and reference electrodes, respectively. The ferrocene/ferrocenium redox couple was used as an external standard. The optimized molecular structures were calculated by density functional theory (DFT) method with the B3LYP functional and the 6-31G(d,p) basis set using a modelling software (Gaussian 16). Grazing-incidence wide-angle X-ray scattering (GIXS) measurements were performed at the Pohang Accelerator Laboratory (beamline 3C, Republic of Korea) with incidence angle of 0.12°.

Nanoparticle (NP) fabrication: The fabrication of nanoparticles was carried out using a mini-emulsion solvent evaporation protocol. Stock solutions were initially prepared by dissolving the requisite polymers and small molecules in chloroform (0.50 mg mL^{-1}), followed by stirring at 45 °C for 60 minutes to promote complete solubilization. Once the donor and acceptor solutions were combined to the specific ratio, a 4 mL portion of this organic phase was added to 8 mL of aqueous TEBS (0.5 wt%). This mixture underwent sonication for 5 minutes to generate a stable emulsion. Subsequently, the organic solvent was driven off by heating the system to 85 °C, yielding an aqueous dispersion of surfactant-stabilized nanoparticles, which was finally filtered through a 0.45 µm glass fiber membrane.

Hydrogen evolution measurement: Hydrogen evolution was monitored in a recirculating reactor system (Agilent 8890 GC). The reaction utilized a 6 mL solution of 0.2 M ascorbic acid containing 10 wt% Pt and 0.5 mg of nanoparticles, initially prepared by mixing 2 mL of NP dispersion with 4 mL of deionized (DI) water. For mass-normalization studies, the NP stock-to-water ratio was varied within the fixed 6 mL total volume. The NP suspensions were loaded into a recirculating batch reactor with an illumination area of 3.9 ± 0.1 cm^2 connected in-line to a gas chromatography. Following five Ar degassing cycles to 500 mbar, the samples were irradiated by a solar simulator (AM1.5G, 100 mW cm^{-2}) while stirring at 700 rpm. H_2 generation was detected using a thermal conductivity detector equipped with a molecular sieve column. The hydrogen evolution rate (HER) was expressed as both area-normalized activity, obtained by dividing by the illumination area (3.9 cm^2), and mass-normalized activity, obtained by dividing by the photocatalyst mass.

External quantum efficiency (EQE) measurement: EQE assessments followed the protocol established for hydrogen evolution, with the exception that monochromatic band-pass filters were utilized. Prior to spectral measurements, the system underwent a 3-hour photodeposition phase under AM 1.5G simulated solar irradiation. Following this activation period, the reactor was evacuated and purged with Argon five times to eliminate any accumulated hydrogen. The light source was then fitted with the appropriate band-pass filters for EQE data collection. The incident photon flux was quantified using a calibrated ThorLabs probe. The EQE values were derived using equation of EQE (%) = $\frac{2n\,\mathrm{H_2}}{n\ \mathrm{photons}} \times\ 100\%$

Organic photovoltaic (OPV) fabrication and characterization: OPVs with a conventional architecture (ITO/poly(3,4-ethylenedioxythiophene):poly(styrenesulfonate)

(PEDOT:PSS)/active layer/2,9-Bis(3-((3-(dimethylamino)propyl)amino)propyl)anthra[2,1,9-def:6,5,10- d'e'f']diisoquinoline-1,3,8,10(2H,9H)-tetraone (PDINN)/Ag) were fabricated following the same procedures described in the previous report.[3] Current density–voltage (*J*–*V*) characteristics were measured using a Keithley 2400 source meter under AM 1.5G illumination (100 mW cm^{-2}) from a solar simulator (K201 LAB55, McScience) that meets Class AAA performance according to ASTM standards. The light intensity was calibrated using a certified silicon reference cell (K801SK302, McScience). The average measurement time per device was 5.9 s. External quantum efficiency (EQE) spectra were recorded using a K3100 IQX system (McScience Inc.) equipped with a monochromator (Newport) and an optical chopper (MC2000, Thorlabs).

Cryo-TEM measurement: Cryo-TEM measurements were carried out using a Thermo Fisher Scientific Titan Krios G3 cryo Transmission Electron Microscope equipped with a Schottky X-FEG high brightness electron source, Cs image corrector for compensation of spherical aberration (Cs) and semi-automated correction of astigmatism, symmetric constant power C-TWIN objective lens with wide pole piece gap of 11 mm, Falcon 3EC pre-GIF direct electron detector camera, and Selectris energy filter coupled to a Falcon 4i direct electron detector camera. All specimens prior to cryo-TEM measurements were prepared using an automated Vitrobot Mark-IV plunge freezer (Thermo Fisher Scientific). A 3.5 μL portion of the nanoparticle dispersion was placed on a carbon-coated Quantifoil MultiA copper TEM grid followed by 3 seconds of blot time and plunge-freezing into liquid ethane cooled by liquid nitrogen. All subsequent handlings of sample grids were performed under cryogenic conditions. Cryo-TEM analysis was performed under an accelerating voltage of 300 kV and acquired at

×215,000 magnification with cumulative electron dose limited to 40 e/Å^2. Image stacks were recorded in counting mode and motion-corrected using Digital Micrograph software (Gatan).

ICP-MS measurement: The concentration of photodeposited Pt was quantified by inductively coupled plasma mass spectrometry (ICP-MS, PerkinElmer NexION 2000B). After photodeposition, the nanoparticle samples were diluted to a nanoparticle concentration of 4 μg mL^{-1} in 2% aqueous HNO3 prepared from concentrated nitric acid and deionized water (18.2 MΩ cm). External calibration was performed using a linear calibration curve constructed from SLS-derived Pt standards, and a QMx mixed standard was used as the certified reference material. All blanks, standards, and samples were spiked with In (1 ng g^{-1}) as an internal standard to correct for instrumental drift. A 2% HNO_3 blank was analyzed together with each batch to determine the background level. The Pt concentration was kept below 500 ppb for all analyzed samples, and the results are reported as Pt concentrations in ppb. The Pt deposition yield was estimated by comparing the ICP-MS-quantified Pt amount with the nominal Pt amount initially introduced for photodeposition (10 wt% relative to the photocatalyst mass).

Steady-state photoluminescence (PL) measurement: Steady-state PL spectra were acquired using an Edinburgh Instruments FLS1000 fluorescence spectrophotometer. The system was configured with a continuous-wave xenon lamp as the excitation source. Signal detection covered both visible and near-infrared (NIR) regions, facilitated by a silicon photomultiplier (PMT-980) and a cryogenically cooled InGaAs detector (InGaAs-1650, liquid nitrogen), respectively. Measurements were performed using discrete excitation wavelengths of 580 and 780 nm.

Ultrafast transient Absorption Spectroscopy (uf-TAS): Ultrafast carrier dynamics were investigated using a fs-TAS system based on an amplified Ti:sapphire laser (Solstice, Spectra Physics; 800 nm, <200 fs, 1 kHz). Excitation pulses were tuned via an optical parametric amplifier (TOPAS Prime) coupled with a frequency mixer (NirUVis), while the probe beam consisted of a white-light continuum generated in a sapphire crystal. This probe covered visible (450–800 nm) or NIR (850–1400 nm) ranges, with time delays up to 6 ns introduced by a mechanical stage. The probe beam was split into signal and reference arms, both detected by multichannel spectrometers (Si or InGaAs sensors). To ensure precision, the pump was modulated at 500 Hz, and pulse energies were quantified using an OPHIR Photonics energy meter (VEGA P/N 7Z01560, 500 μm aperture). Samples were prepared to an optical density of 0.6 at the excitation wavelength and measured under argon.

Microsecond Transient Absorption Spectroscopy (ms-TAS): Long-lived transient species were probed using a nanosecond Nd:YAG laser (OPOTEK Opolette 355 II; 6 ns pulses, 355 nm) capable of tunable output (410–2200 nm). The probe light, provided by a 100 W quartz halogen lamp, was passed through the sample and analyzed via a monochromator and Si photodiode (Hamamatsu S1722-01). Data collection was triggered by scattered laser light, with appropriate long-pass filters installed to reject pump scatter. A custom LabVIEW interface managed dual-timescale acquisition: microsecond-to-millisecond signals were amplified (Costronics 2011) and recorded on an oscilloscope (Tektronix DPO 3012), whereas millisecond-to-second signals were acquired directly via a DAQ card (National Instruments USB-6361). Samples were adjusted to an absorbance of 0.6 and maintained under an argon atmosphere.

Photoinduced Absorption Spectroscopy (PIA): Steady-state photoinduced absorption was measured on a custom-built apparatus. The excitation source was a high-power LED regulated by a DC supply (TTi QL564P) and pulsed via a MOSFET transistor (STMicroelectronics STF8NM50N). Control and direct signal acquisition (without pre-amplification) were handled by a National Instruments USB-6361 DAQ card. Excitation fluences were calibrated using a Thorlabs PM100 meter with an S120UV sensor. Kinetic measurements utilized 530 nm (3 mW cm^{-2}) excitation. All nanoparticle suspensions were characterized in an argon environment.

Supporting Figures & Tables

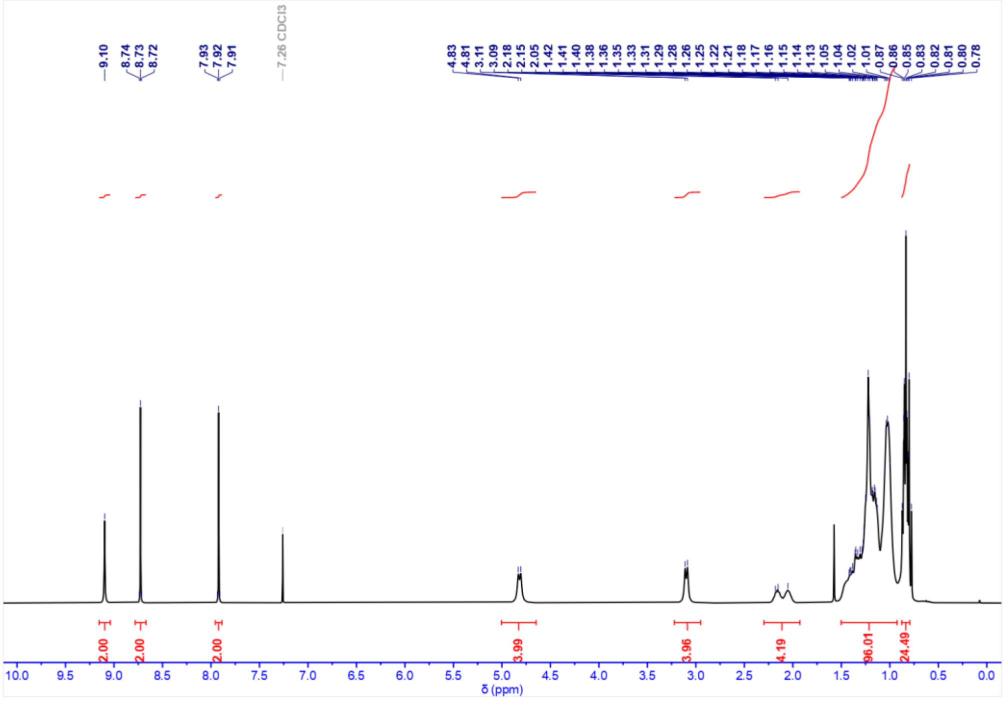

Figure S1. ^{1}H-NMR of MY in $CDCl_3$. ^{1}H NMR (500 MHz, $CDCl_3$) δ 9.10 (s, 2H), 8.73 (s, 2H), 7.92 (s, 2H), 4.82 (d, J = 7.8 Hz, 4H), 3.10 (d, J = 7.4 Hz, 4H), 2.22 – 1.94 (m, 4H), 1.43 – 0.98 (m, 96H), 0.90 – 0.75 (m, 24H).

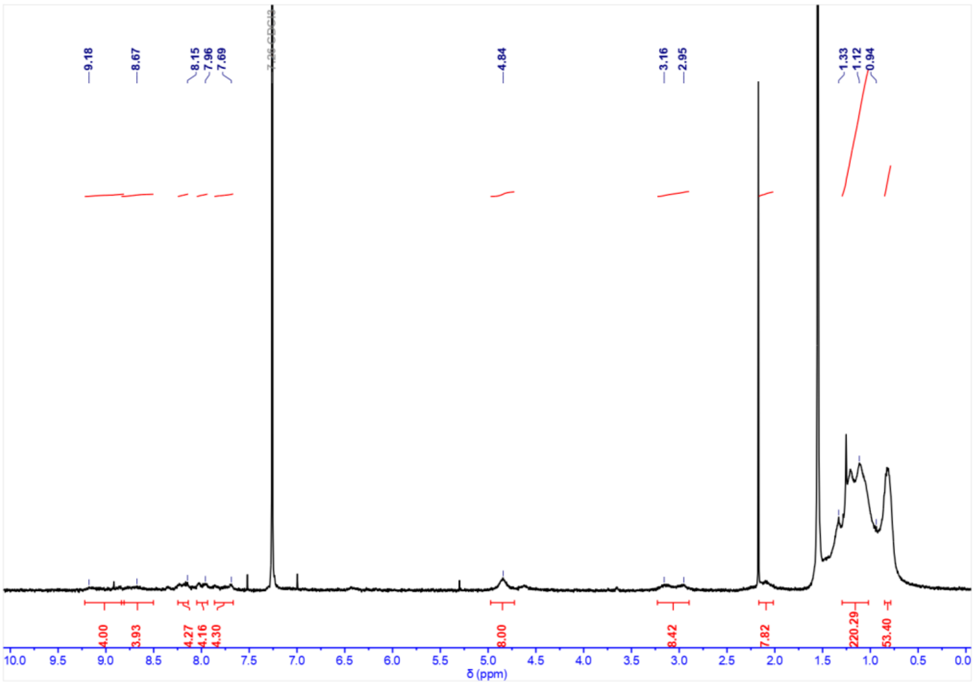

Figure S2 ^{1}H-NMR of DY1 in $CDCl_3$. ^{1}H NMR (500 MHz, $CDCl_3$) δ 9.21-8.82 (m, 4H), 8.81-8.56 (m, 4H), 8.25-8.13 (m, 4H), 8.05-7.92 (m, 4H), 7.86-7.67 (m, 4H), 4.92-4.85 (m, 8H), 3.37-2.72 (m, 8H), 2.38-1.95 (m, 8H), 1.47-0.94 (m, 192H), 0.92-0.76 (m, 48H).

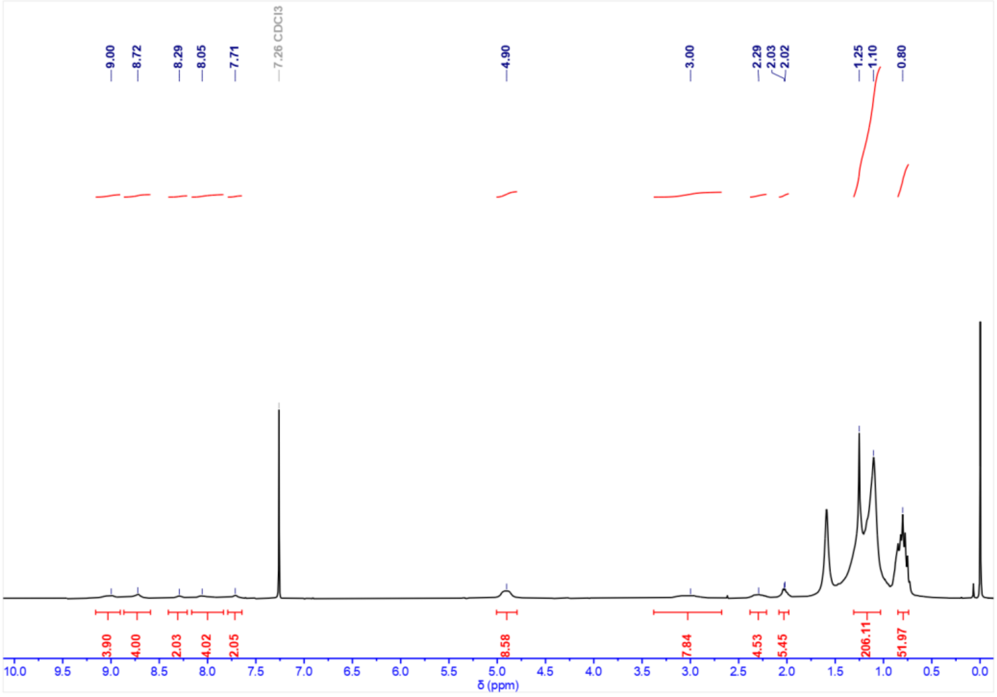

Figure S3 ^{1}H-NMR of DY2 in $CDCl_3$. ^{1}H NMR (500 MHz, $CDCl_3$) δ 9.16-8.91 (m, 4H), 8.86-8.58 (m, 4H), 8.42-8.22 (m, 2H), 8.17-7.83 (m, 4H), 7.78-7.64 (m, 2H), 5.02-4.85 (m, 8H), 3.35-2.70 (m, 8H), 2.38-2.18 (m, 4H), 2.11-1.95 (m, 4H), 1.45 – 0.95 (m, 192H), 0.90 – 0.75 (m, 48H).

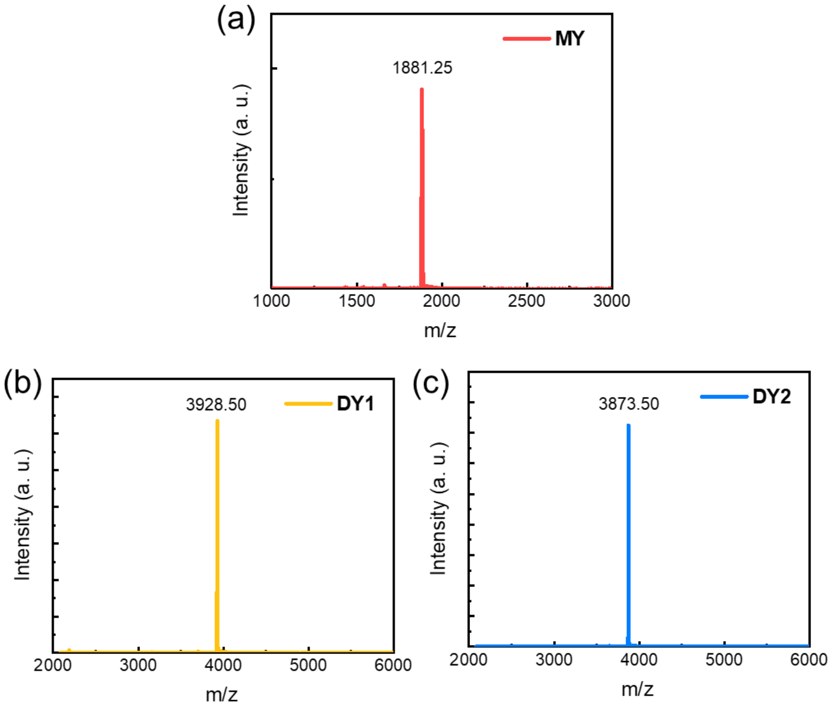

Figure S4. MALDI-ToF spectra of (a) MY, (b) DY1, and (c) DY2.

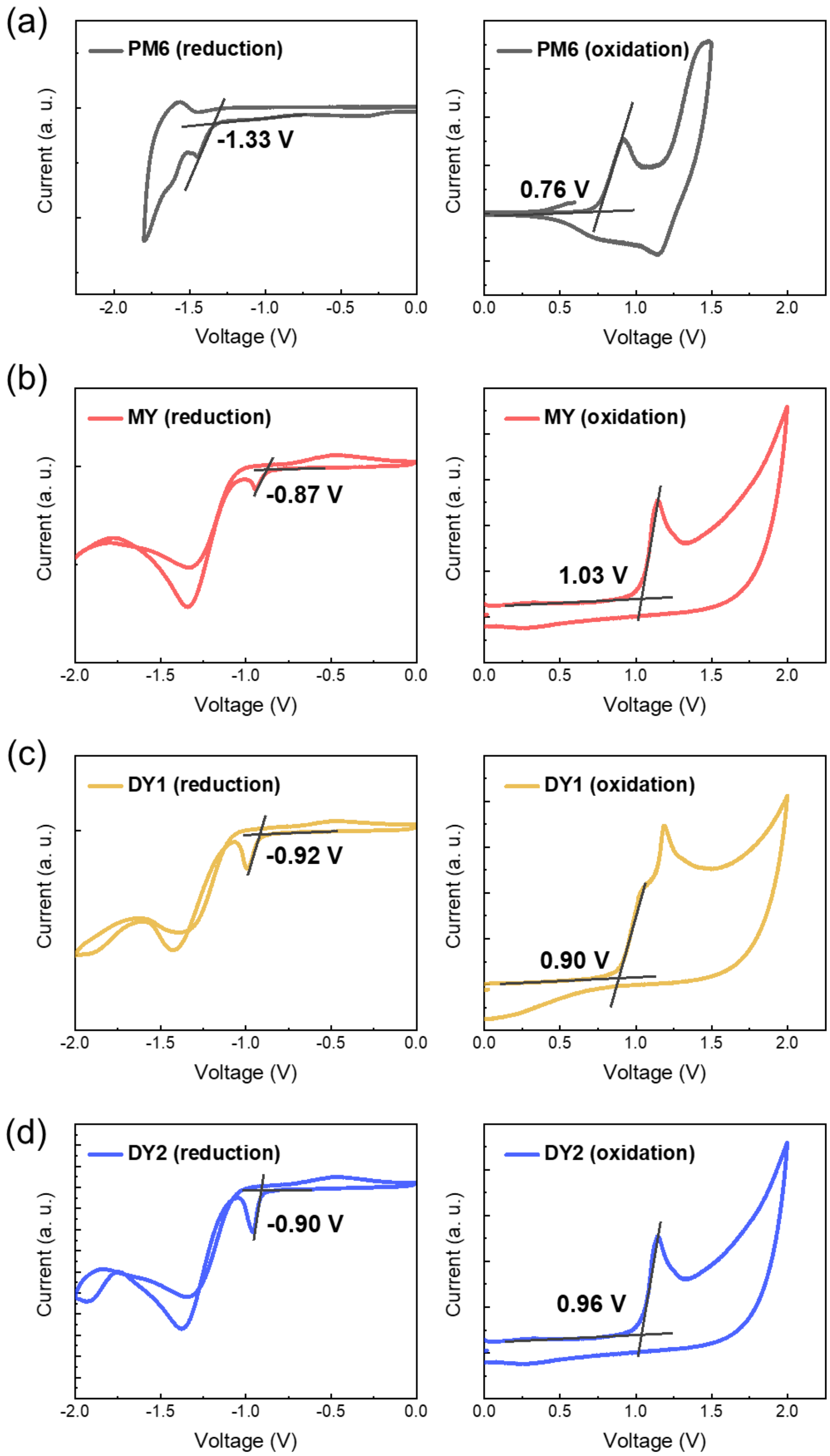

Figure S5. Cyclic voltammograms of (a) PM6, (b) MY, (c) DY1, and (d) DY2.

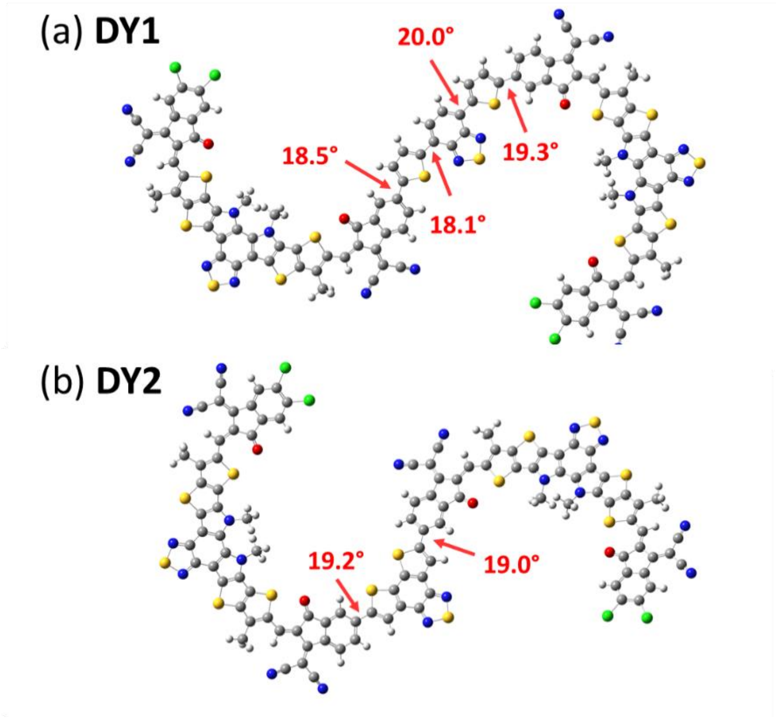

Figure S6. Optimized molecular conformations and dihedral angles of the (a) DY1 and (b) DY2 obtained from DFT simulations.

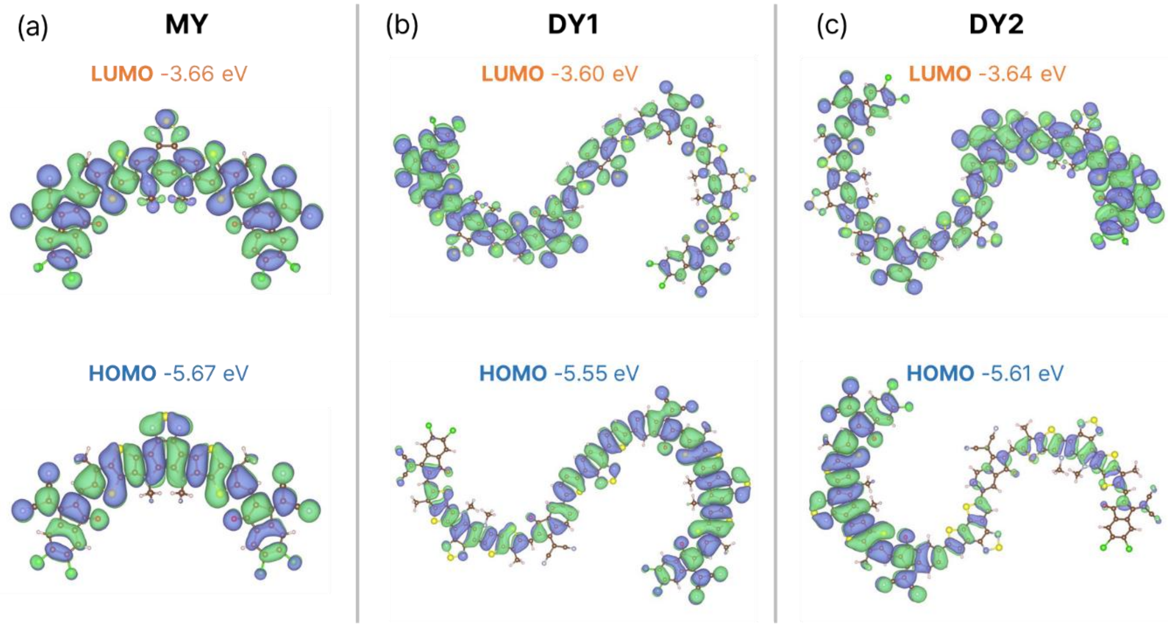

Figure S7. DFT-calculated HOMO and LUMO distributions and corresponding energy levels of (a) MY, (b) DY1, and (c) DY2.

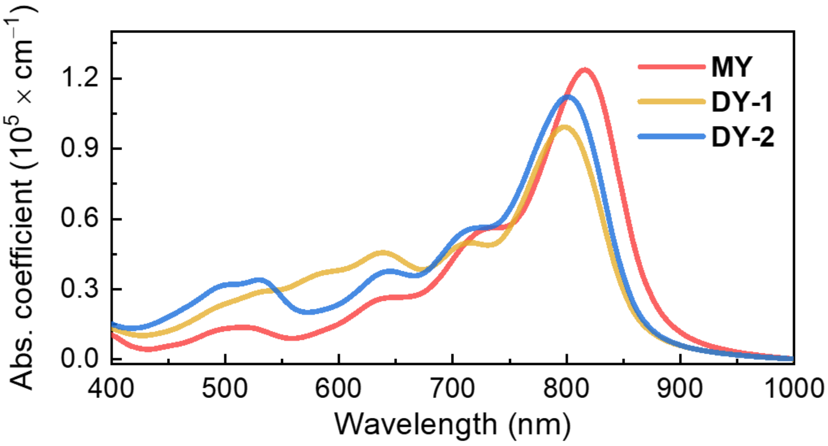

Figure S8. Absorption coefficient of neat acceptor films spin-coated using chloroform solvent.

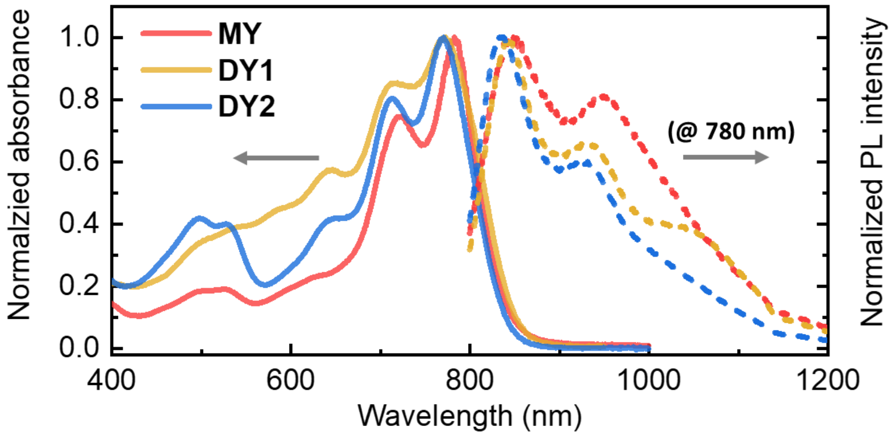

Figure S9. UV–vis absorption and PL emission spectra of pristine acceptor NPs. PL spectra were recorded under excitation at 780 nm.

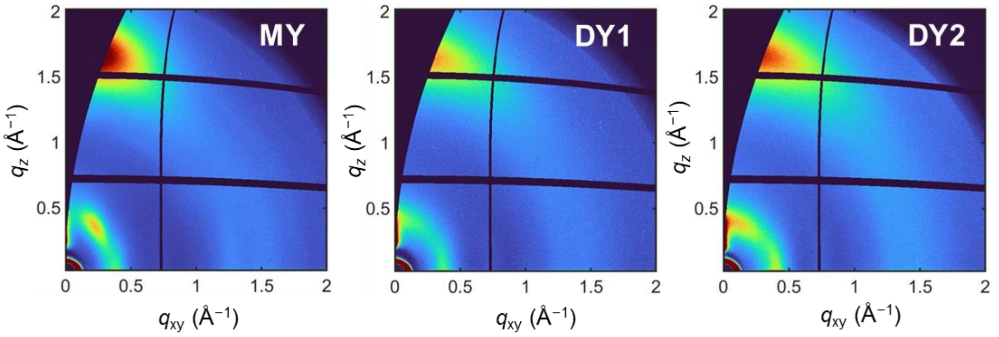

Figure S10. GIXS 2D-images of MY, DY1, and DY2 films.

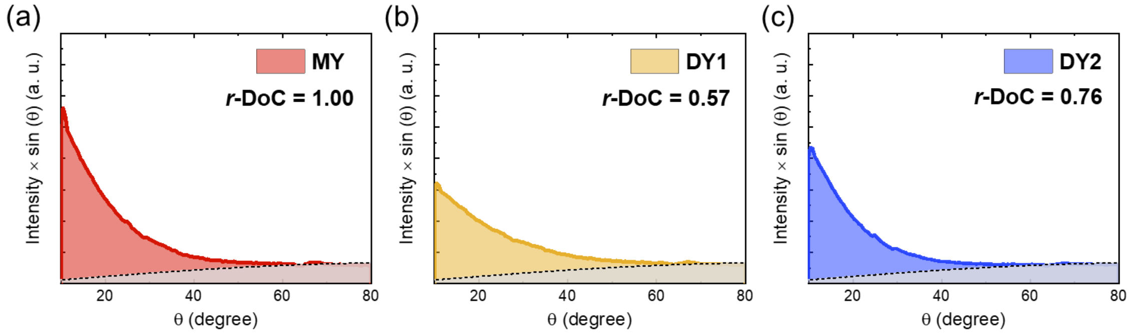

Figure S11. Pole figures and estimated relative degree of crystallinity (*r*-DoC) of (a) MY, (b) DY1, and (c) DY2 extracted from the GIXS patterns at the (010) scattering peak region (q = 1.5–1.8 Å^{-1}).

Table S1. Electron mobility of acceptor films measured by the space-charge-limited current (SCLC) method.

System	μ_e^{SCLC} (cm V^{-1} s^{-1})[a]
**MY**	4.8×10^{-4}
**DY1**	7.8×10^{-5}
**DY2**	2.7×10^{-4}

[a] Average values obtained from three independently fabricated devices.

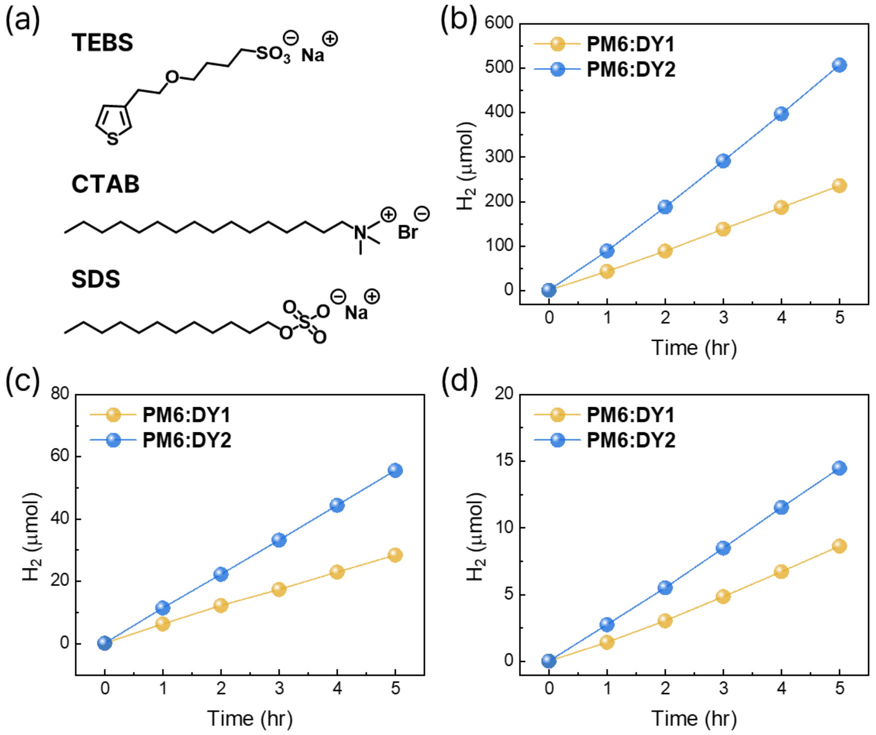

Figure S12. (a) Molecular structures of the surfactants TEBS, CTAB, and SDS. (b–d) H_2 evolution over time for PM6:DY1- and PM6:DY2-based OPCs (blend ratio = 1:1) prepared with different surfactants at a donor:acceptor blend ratio of 1:1 and a photocatalyst concentration of 0.08 mg mL^{-1}: (b) TEBS, (c) CTAB, and (d) SDS.

Table S2. Hydrogen evolution rates of PM6:DY1 and PM6:DY2 NPs (blend ratio = 1:1) prepared with different surfactants under identical AA and Pt loading conditions.

Surfactant	System	HER (μmol h^{-1} cm^{-2})
**TEBS**[a]	PM6:DY1	11.9
	PM6:DY2	25.3
**CTAB**[b]	PM6:DY1	7.1
	PM6:DY2	13.8
**SDS**[b]	PM6:DY1	2.1
	PM6:DY2	3.6

Each surfactant was used at a concentration of [a]0.05 mg mL^{-1} and [b]0.005 mg mL^{-1}.

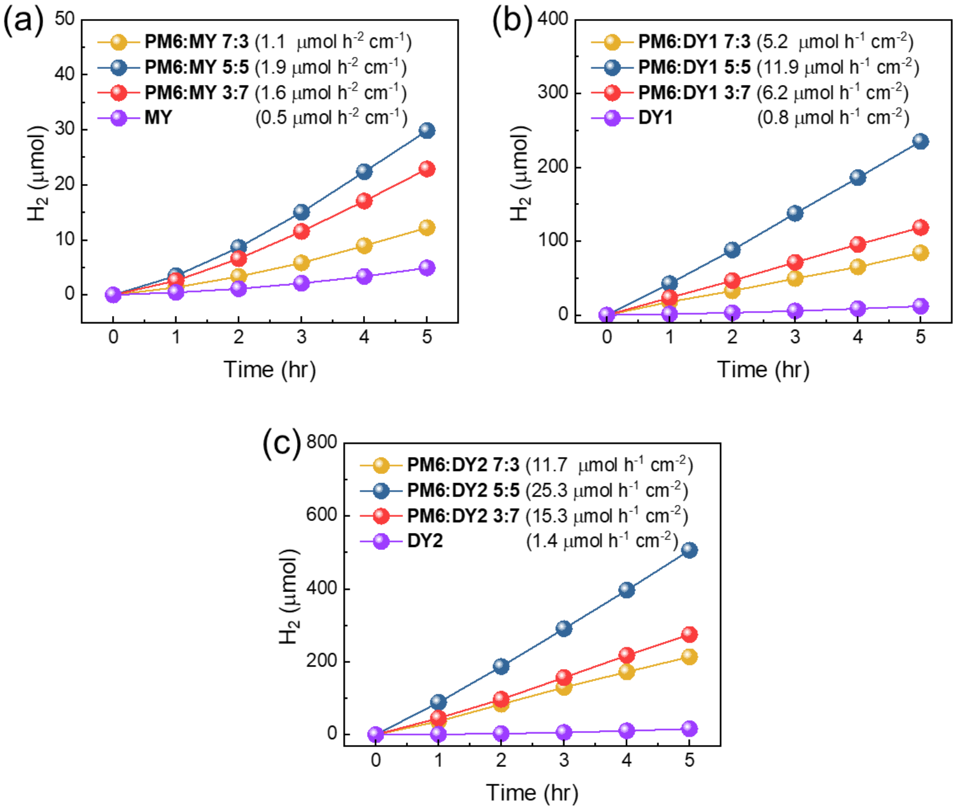

Figure S13. H_2 evolution over time of (a) PM6:MY, (b) PM6:DY1, and (c) PM6:DY2-based OPCs at different blend ratios. (0.5 mg of photocatalyst in 6 mL solution, 0.2 M AA, and 10 wt% Pt co-catalyst)

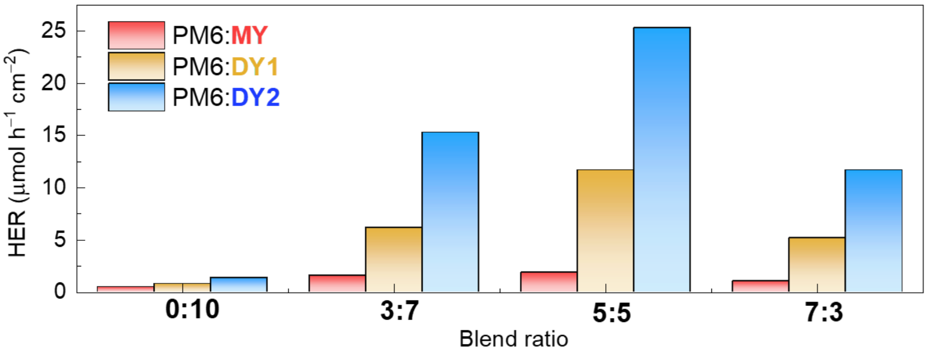

Figure S14. HER per illumination area of the OPCs depending on acceptor types and blend ratios (PM6:acceptor).

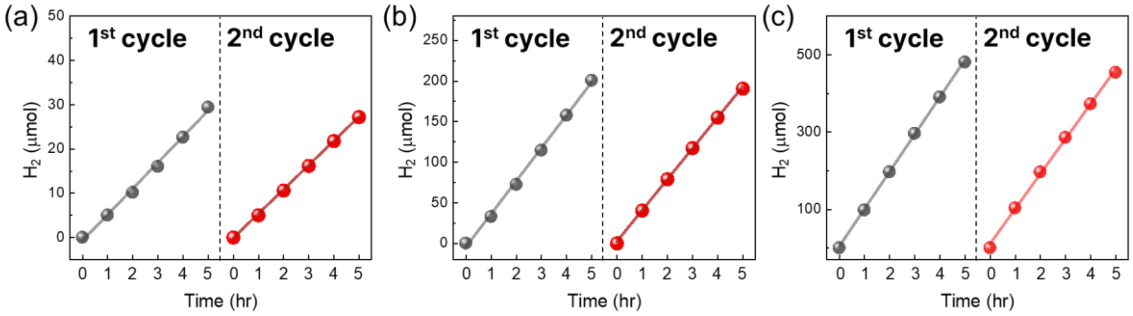

Figure S15. Repeated-cycle H_2 evolution profiles of OPCs based on (a) PM6:MY, (b) PM6:DY1, and (c) PM6:DY2 at a 1:1 blend ratio. For the second cycle, the NPs were collected by centrifugation, washed to remove residual AA and oxidized AA, and then redispersed in a fresh AA-containing aqueous solution.

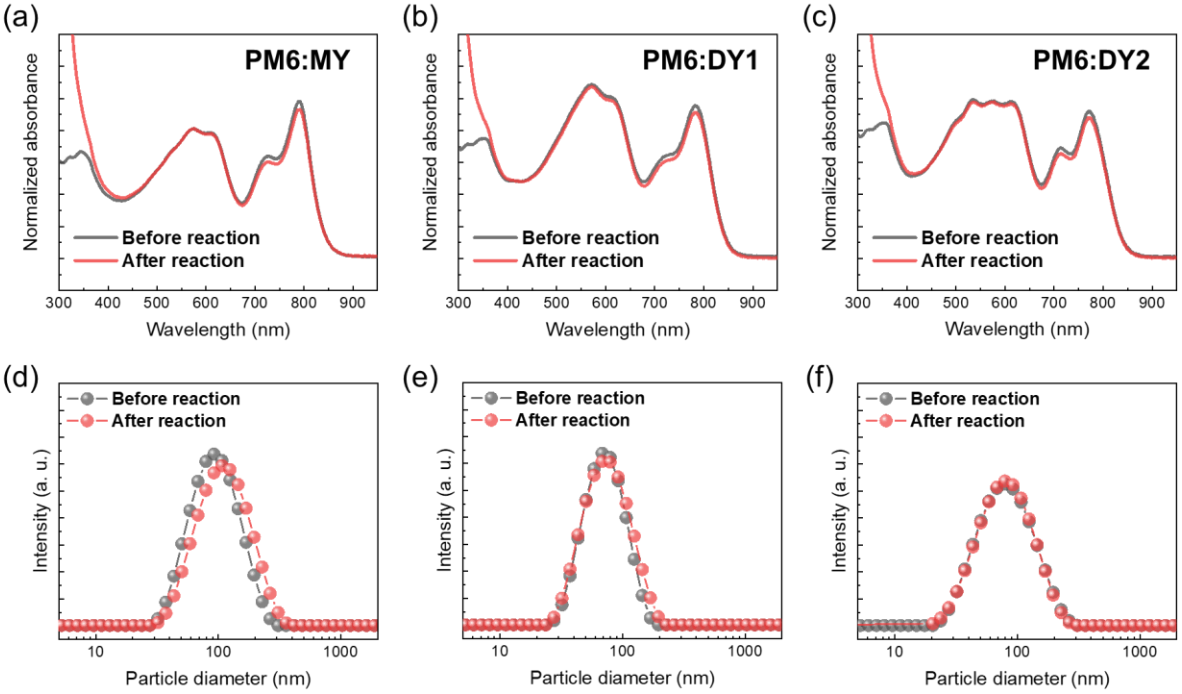

Figure S16. (a–c) Normalized UV–vis absorption spectra and (d–f) DLS size distributions of (a,d) PM6:MY, (b,e) PM6:DY1, and (c,f) PM6:DY2 nanoparticles measured before and after 5 h of photocatalytic H_2 evolution reaction.

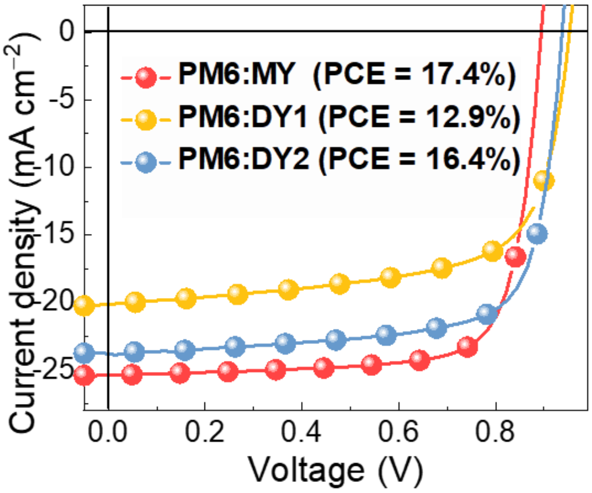

Figure S17. Current density-voltage curves of PM6:acceptor-based OPVs.

Table S3. Photovoltaic parameters of PM6:acceptor-based OPVs.

**Active system**	V_{oc} **(V)**	J_{sc} **(mA cm^{-2})**	**FF**	**PCE$_{max\ (avg.)}$^{a} (%)**
**PM6:MY**	0.89	25.40	0.77	17.38 (17.04)
**PM6:DY1**	0.95	20.13	0.67	12.86 (12.47)
**PM6:DY2**	0.94	23.81	0.74	16.42 (16.03)

[a]Averaged values from 10 independent OPV devices.

Table S4. Comparison of HER performance of OPCs in the literature and this study (normalized by photocatalyst mass).

Year	OPC system	Concentration (mg mL^{-1})	HER (mmol g^{-1} h^{-1})	Ref
**2016**	PFBT	0.019	4	[2]
**2017**	PFODTBT	0.019	12	[3]
	CTF-2	1	0.4	[4]
**2018**	F8BT	0.133	0.2	[5]
	L-PyBT	0.5	1.7	[6]
	PFTFQ-PtPy15	-[b]	10.2	[7]
**2019**	PFODTBT[c]	0.023	12	[8]
	HE(e)-CPDOT	0.03	0.8	[9]
	PyPm	0.5	0.4	[10]
	P1	0.5	1.0	[11]
	PBDTBT-7EO	0.05	16	[12]
**2020**	PDBTSO	0.1	33.8	[13]
	PFTBTA-PtPy	0.06	7.3	[14]
	PCDTBT:PC_{61}BM	–	105	[15]
	PTB7-Th:EH-IDTBR	0.1	64	[16]
**2021**	PFTBTA:NCADs	0.2	4.8	[17]
	PFO	0.02	1.2	[18]
	PTTPA:PFTBTA	–	43.9	[19]
	PSO-FNBr	0.025	14.5	[20]
	PS-PEG5	0.2	28.8	[21]
	PFBT:PFODTBT:ITIC	0.03	61	[22]
**2022**	PN-2	0.8	6.7	[23]
	F8BT-BFPA	0.02	11	[24]
	Y6	0.075	4.2	[25]
	PM6:Y6	0.15	8	[26]
	PG6	0.05	5.8	[27]
	PM6:Y6	0.083	44	[28]
	PM6:PCBM	0.083	71.9	
	gIDTBT:oIDTBR	0.083	18.5	[29]
	PBDB-T:ITIC	0.05	257	[30]
	PM6:TPP	0.066	82	[31]
	F1	0.016	152	[32]
**2023**	P(BTOEGL-2F2T)	0.02	8.9	[33]
		0.02	7.6	
	Y5	0.04	22	[34]
	POZ-M:ITIC	0.045	63	[35]
	PFODTBT:ITIC	0.030	28	[36]
	PM6:ITCC-M:IDMIC-4F	0.05	307	[37]
	PM6:Y6-2OH	0.083	102	[38]
	Y6-S	0.003	217	[39]

	PM6:Y6CO	0.003	320	[40]
**2024**	TBT-3	0.3	0.1	[41]
	PFBT	0.02	3.2	[42]
	PIDT-T8BT:Y6	0.086	6	[43]
	PITIC-ThF	0.5	55.8	[44]
**2025**	hPTB7-Th:PCBM	0.05	111.5	[45]
	PF$_g$BTSO-T25	0.025	209	[46]
	as-DCPIC	0.02	11.88	[47]
	PM6:2FBP-4F (TEBS)	0.0017	562	[48]
	PM6: 2FBP-4F (DTAB)	0.0067	946.1	[48]
**2026**	IT-PMI	0.023	112.7	[49]
	PM6:DY2	0.0083	564.2	**This study**

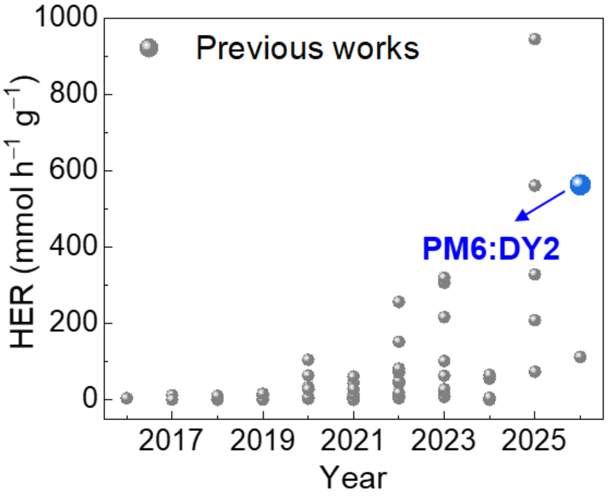

Figure S18. Comparison of the HER per unit mass of photocatalyst for PM6:DY2 alongside other reported systems.

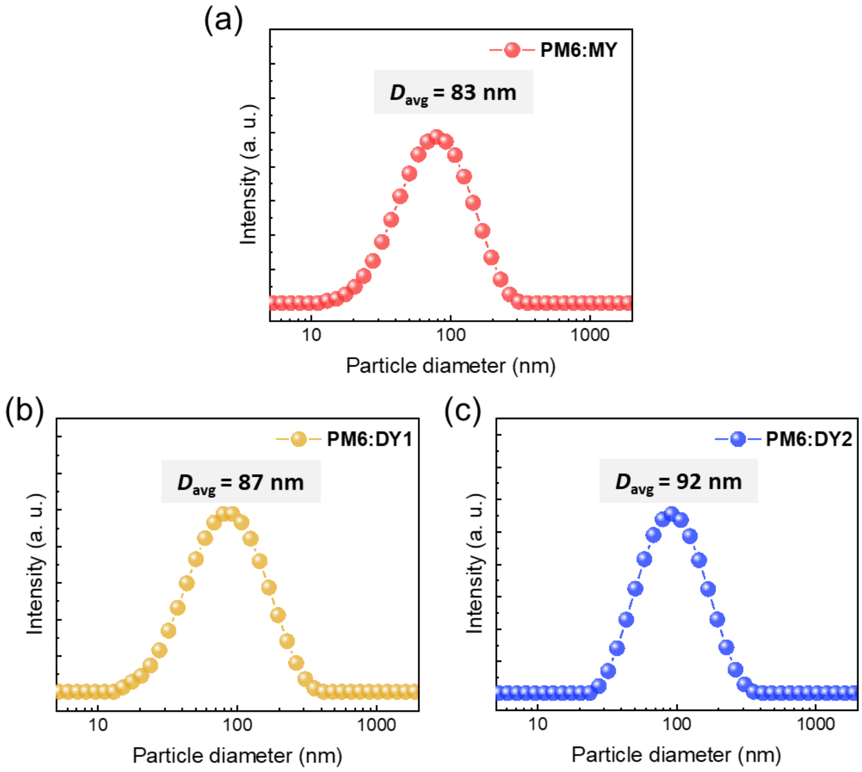

Figure S19. DLS profiles of (a) PM6:MY, (b) PM6:DY1, and (c) PM6:DY2 NPs at 1:1 blend ratio. The average particle diameter was obtained from 30 measurements.

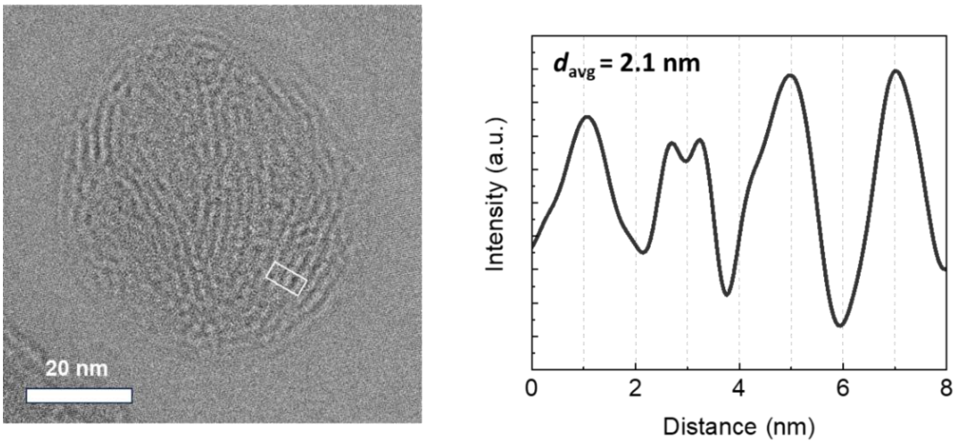

Figure S20. Cryo-TEM image of neat PM6 NPs and intensity profiles of the periodic spacings corresponding to the region highlighted in the rectangle.

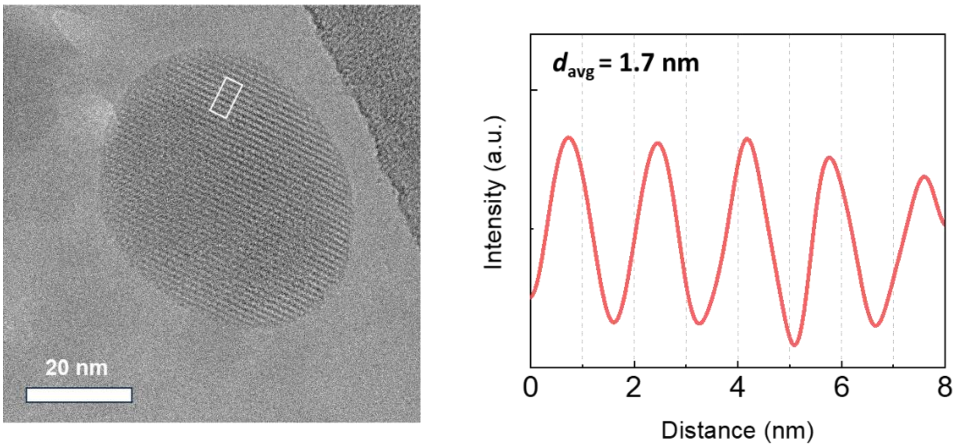

Figure S21. Cryo-TEM image of neat MY NPs and intensity profiles of the periodic spacings corresponding to the region highlighted in the rectangle.

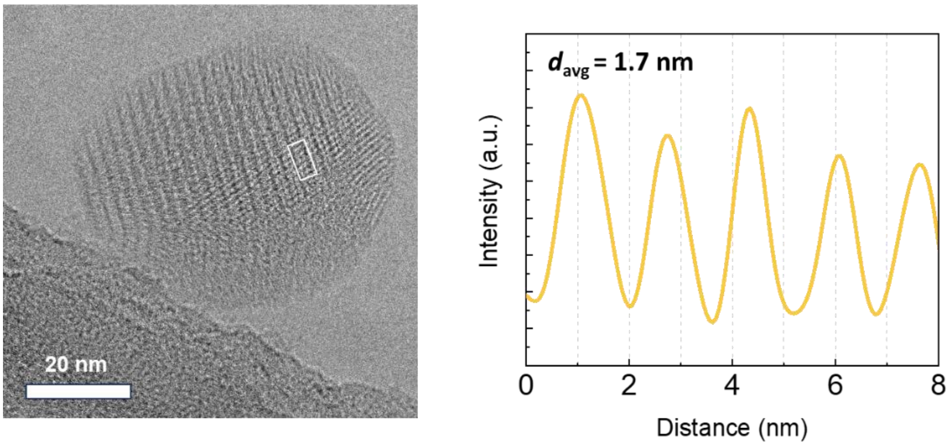

Figure S22. Cryo-TEM image of neat DY1 NPs and intensity profiles of the periodic spacings corresponding to the region highlighted in the rectangle.

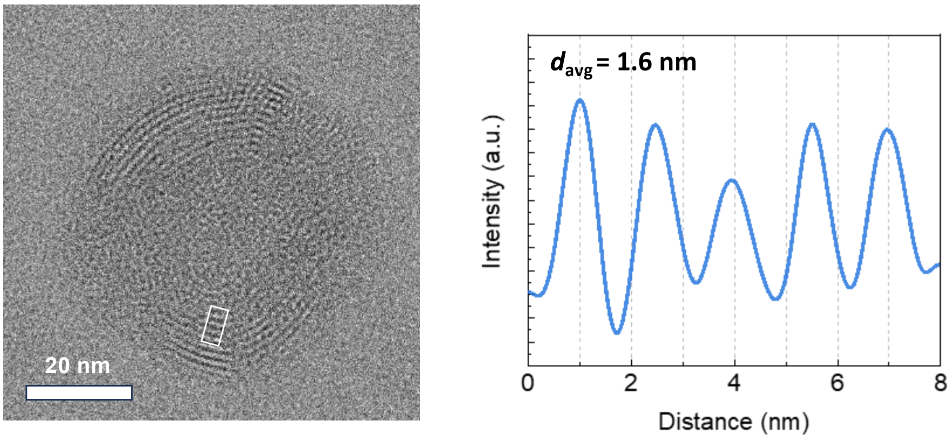

Figure S23. Cryo-TEM image of neat DY2 NPs and intensity profiles of the periodic spacings corresponding to the region highlighted in the rectangle.

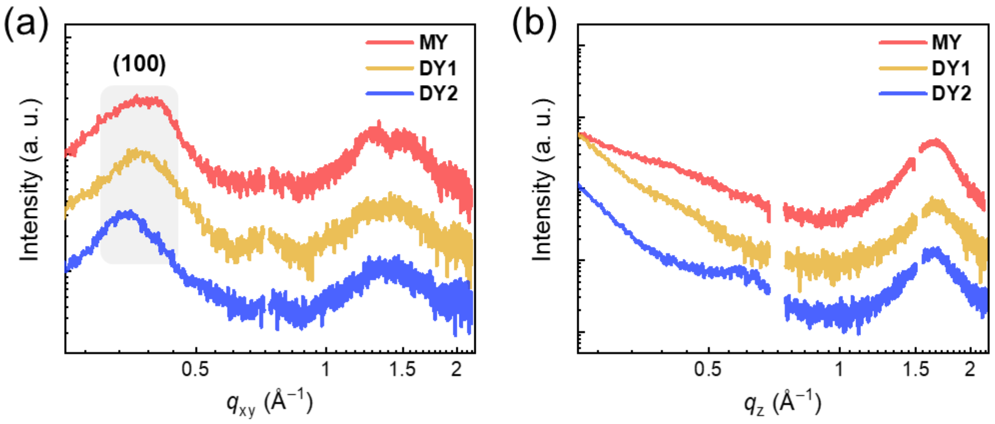

Figure S24. GIXS linecut profiles of neat acceptor films in the (a) in-plane and (b) out-of-plane directions.

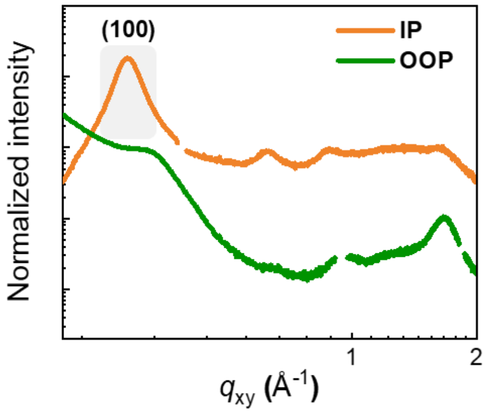

Figure S25. GIXS linecut profiles of neat PM6 film in the in-plane (IP) and out-of-plane (OOP) directions.

Table S5. q_{xy} values and calculated lamellar spacings of pristine constituent films, derived from GIXS linecut profiles of the (100) scattering peaks in the IP direction.

Material	$q_{xy\,(100)}{}^{IP}$ (Å^{-1})[a]	*d*-spacing (nm)[b]
**MY**	0.38	1.7
**DY1**	0.37	1.7
**DY2**	0.37	1.7
**PM6**	0.29	2.2

[a]Estimated from GIXS linecut profiles in the IP direction. [b] *d*-spacing calculated as $2\pi/q_{xy\,(100)}{}^{IP}$ from the (100) peak in the IP direction, corresponding to the lamellar spacing.

Table S6. Pt concentration and estimated photodeposition yield in PM6:acceptor nanoparticles determined by ICP-MS.

System	Pt concentration (ppb)[a]	Deposition yield (%)[b]
**QCC Standard (180 ppb)**	179.3	–
**PM6:MY**	22.1	27.6
**PM6:DY1**	35.5	44.4
**PM6:DY2**	39.2	49.0

[a]NP dispersions were diluted to 4 μg mL^{-1} in 2% nitric acid prior to ICP-MS analysis. [b] Estimated based on the nominal Pt loading for photodeposition (10 wt% relative to the photocatalyst mass).

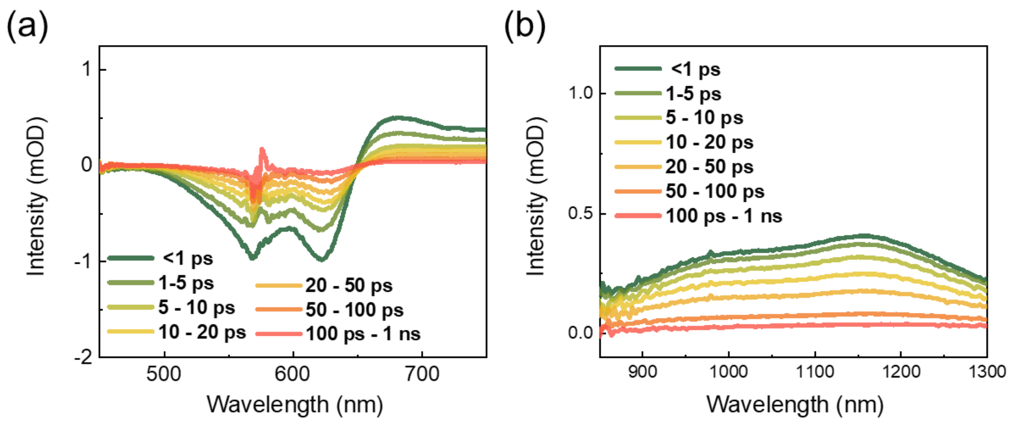

Figure S26. uf-TAS spectra of neat PM6 NPs in the (a) visible and (b) NIR ranges, excited at 580 nm with a fluence of 10 μJ cm^{-2}.

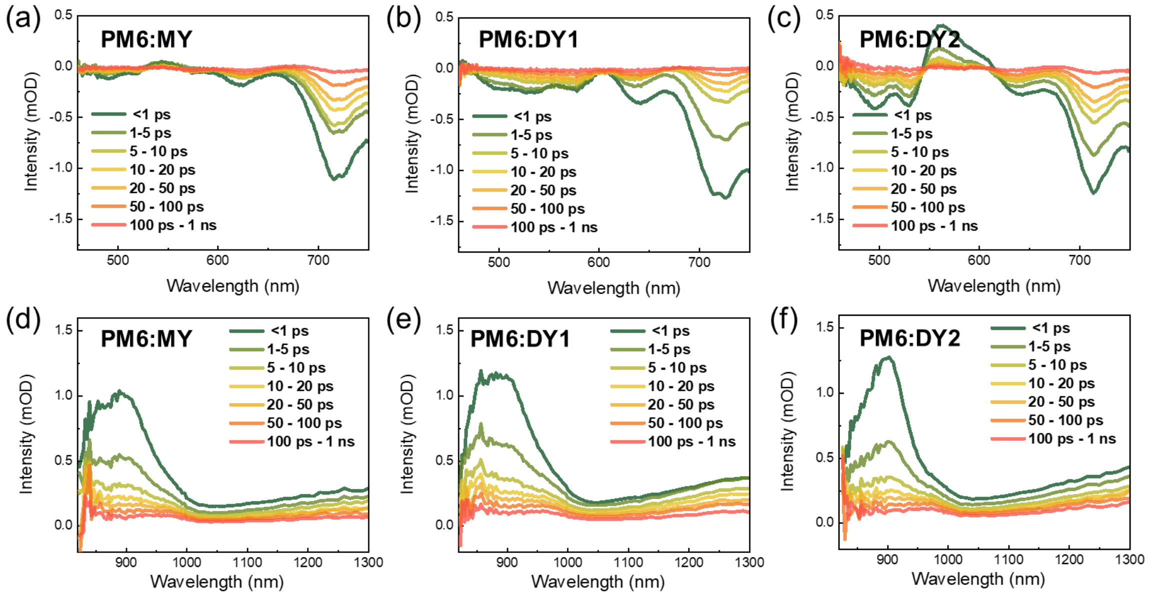

Figure S27. uf-TAS spectra of PM6:acceptor NPs in the visible and NIR ranges for (a, d) PM6:MY, (b, e) PM6:DY1, and (c, f) PM6:DY2, excited at 780 nm with a fluence of 30 μJ cm^{-2}.

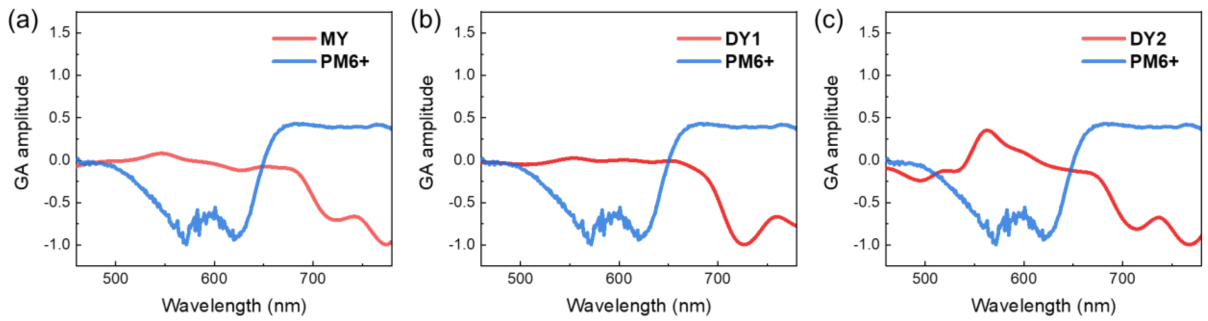

Figure S28. Deconvoluted TAS spectra in the visible wavelength range (450–800 nm) obtained *via* global analysis. The measurements were performed on PM6:acceptor NPs under 780 nm excitation at a fluence of 30 μJ cm^{-2}.

Table S7. Biexponential fitting parameters for the uf-TAS decay traces shown in Figure 4b.

System	A_1	τ_1 (ps)	A_2	τ_2 (ps)	τ_{avg} [a] (ps)
**PM6:MY**	1.4×10^{-4}	5.1	8.2×10^{-5}	374	141
**PM6:DY1**	2.3×10^{-4}	4.4	1.5×10^{-4}	294	119
**PM6:DY2**	1.8×10^{-4}	5.0	1.2×10^{-4}	600	243

[a]Average lifetime calculated using $\tau_{avg} = (A_1\tau_1 + A_2\tau_2)/(A_1 + A_2)$

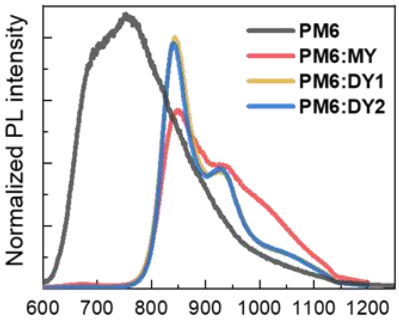

Figure S29. Normalized steady-state PL spectra of PM6:acceptor NPs under 580 nm excitation. The spectra are normalized to the number of absorbed photons by dividing the intensity by $1-10^{A}$ (where A = absorbance at 580 nm).

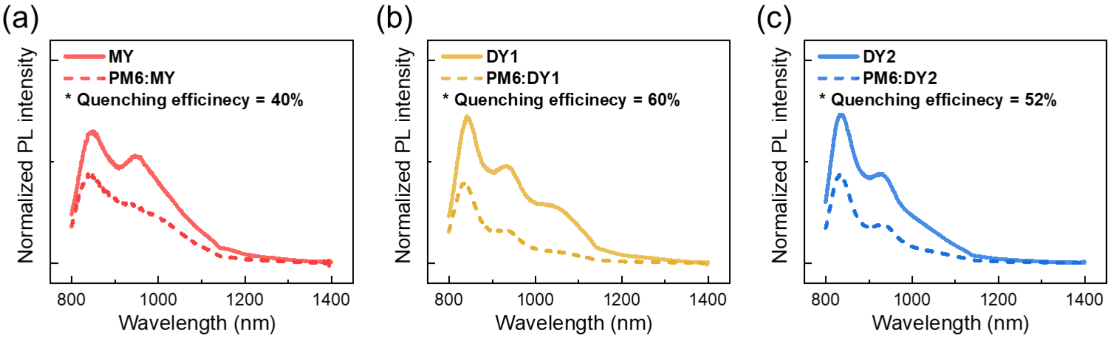

Figure S30. Normalized steady-state PL spectra of (a) PM6:MY, (b) PM6:DY1, and (c) PM6:DY2 NPs under 780 nm excitation. The spectra are normalized to the number of absorbed photons by dividing the intensity by $1-10^{A}$ (where A = absorbance at 780 nm).

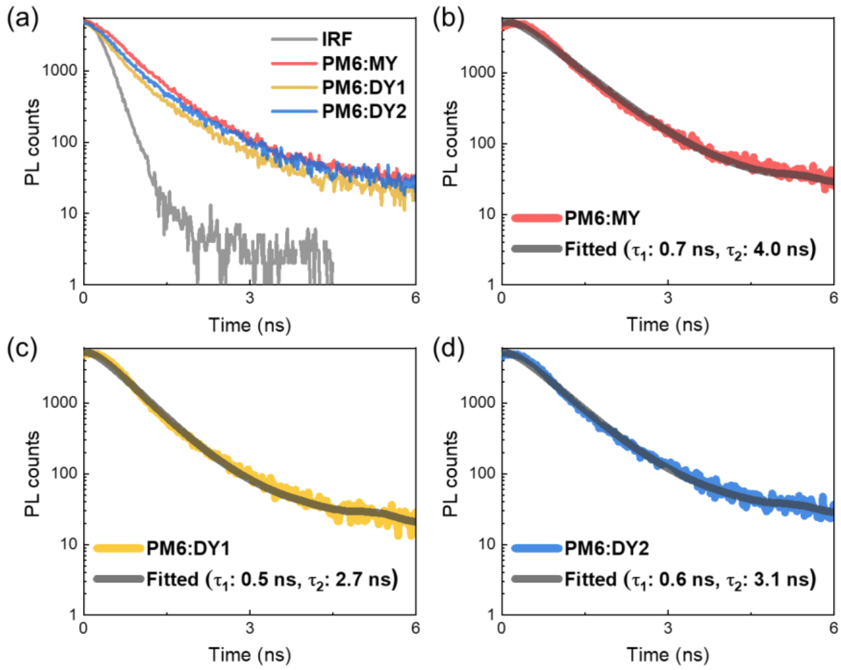

Figure S31. (a) TRPL decay profiles of PM6:acceptor NPs and the instrument response function (IRF). (b–d) Experimental and fitted TRPL decays for (b) PM6:MY, (c) PM6:DY1, and (d) PM6:DY2 NPs. The measurements were performed with excitation at 670 nm and emission monitored at 940 nm.

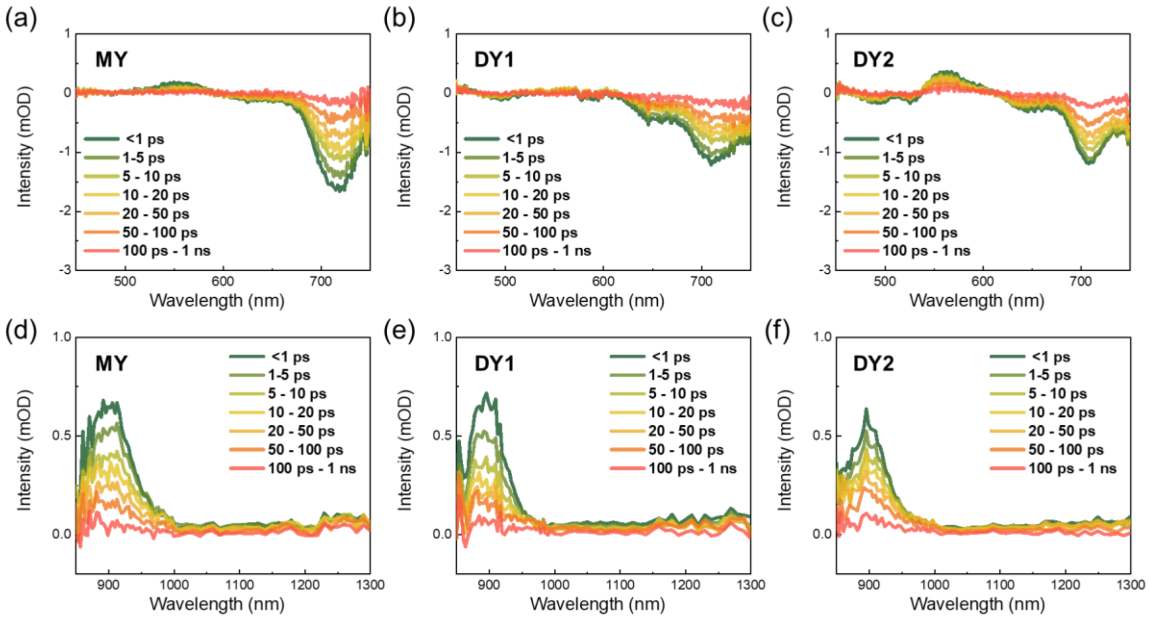

Figure S32. uf-TAS spectra of neat acceptor NPs in the visible and NIR ranges for (a, d) MY, (b, e) DY1, and (c, f) DY2, excited at 780 nm with a fluence of 13 μJ cm^{-2}.

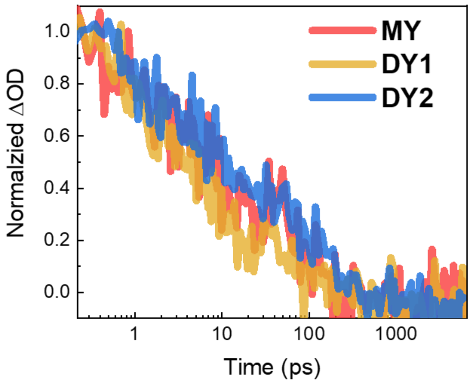

Figure S33. uf-TAS kinetics of neat acceptor NPs excited at 780 nm with a fluence of 13 μJ cm^{-2} and probed at 900 nm, where the signal is assigned to exciton species.

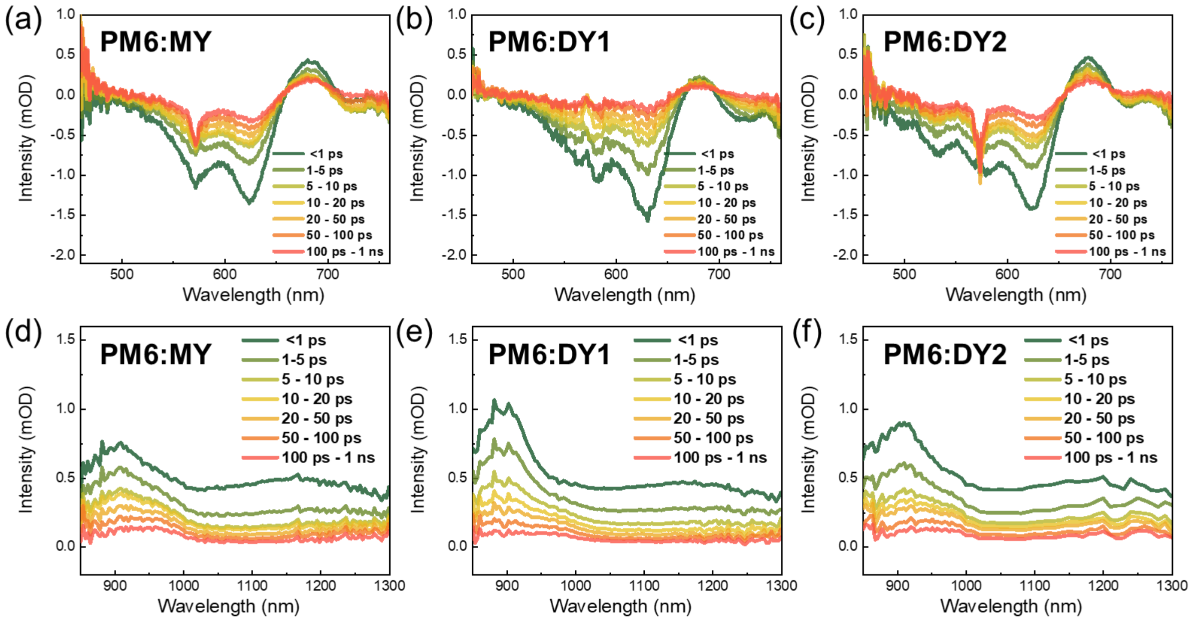

Figure S34. uf-TAS spectra of PM6:acceptor NPs in the (a-c) visible and (d-f) NIR regions: (a, d) PM6:MY, (b, e) PM6:DY1, and (c, f) PM6:DY2. Measurements were performed under 580 nm excitation at a fluence of 30 μJ cm^{-2}.

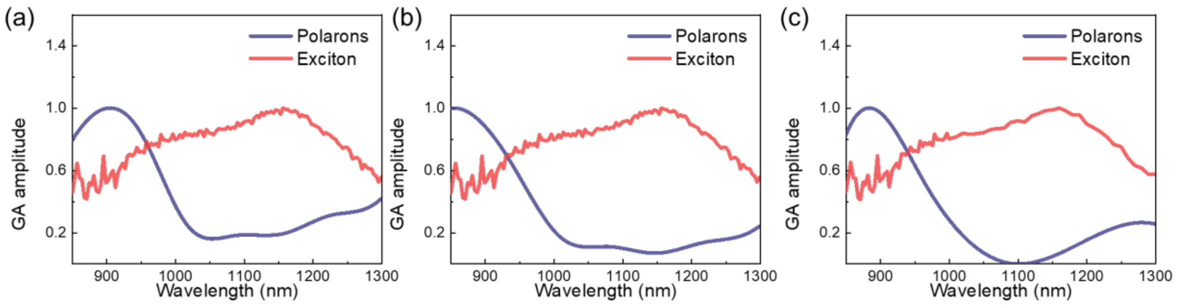

Figure S35. Deconvoluted TAS spectra in the NIR region (850–1300 nm) obtained by global analysis. The measurements were performed on PM6:acceptor NPs under 580 nm excitation at a fluence of 30 μJ cm^{-2}.

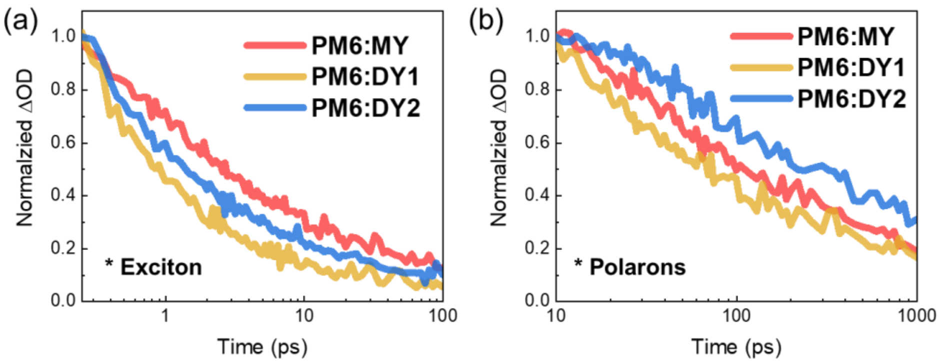

Figure S36. Deconvoluted uf-TAS kinetics of the (a) exciton and (b) polaron components, obtained from global analysis of the NIR region, for PM6:acceptor NPs under 580 nm excitation at a fluence of 30 μJ cm^{-2}.

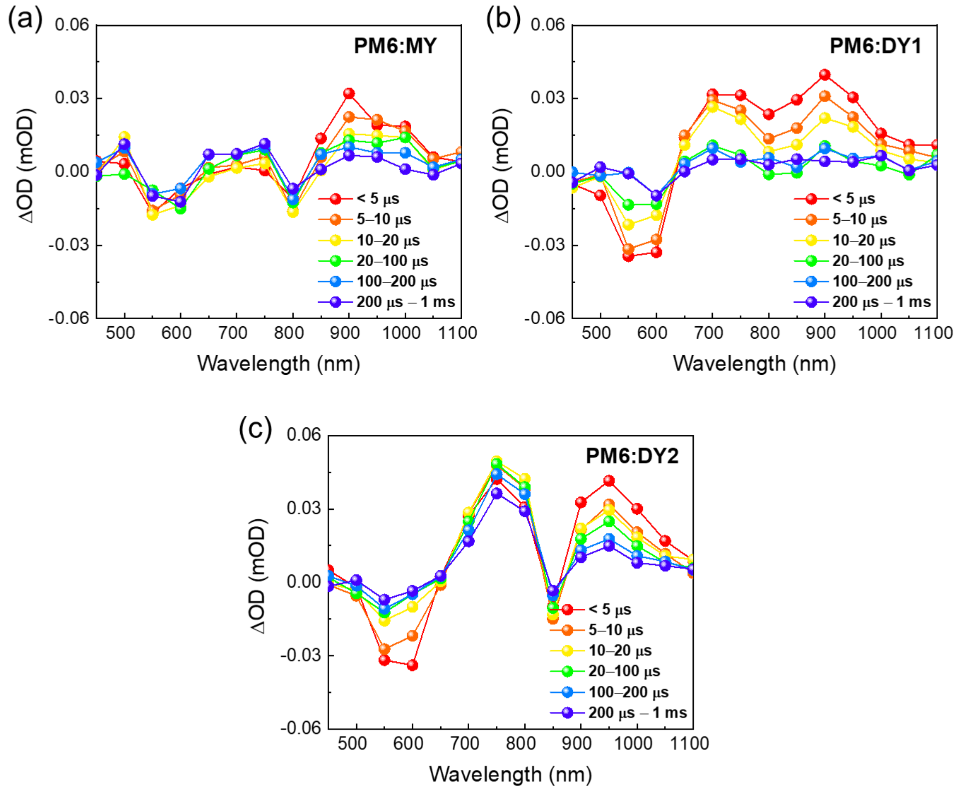

Figure S37. MS-TAS signal intensities of (a) PM6:MY, (b) PM6:DY1, and (c) PM6:DY2 NPs, measured at different time scales and recorded at various probe wavelengths.

Table S8. Biexponential fitting parameters for the MS-TAS decay traces shown in Figure 4c.

**System**	A_1	τ_1 (μs)	A_2	τ_2 (ms)	τ_{avg}[a] (ms)
**PM6:MY**	1.2×10^{-7}	0.14	5.6×10^{-6}	0.52	0.51
**PM6:DY1**	1.7×10^{-7}	0.18	8.2×10^{-6}	1.27	1.24
**PM6:DY2**	2.9×10^{-7}	0.21	1.2×10^{-5}	1.50	1.46

[a]Average lifetime calculated using $\tau_{avg} = (A_1\tau_1 + A_2\tau_2)/(A_1 + A_2)$

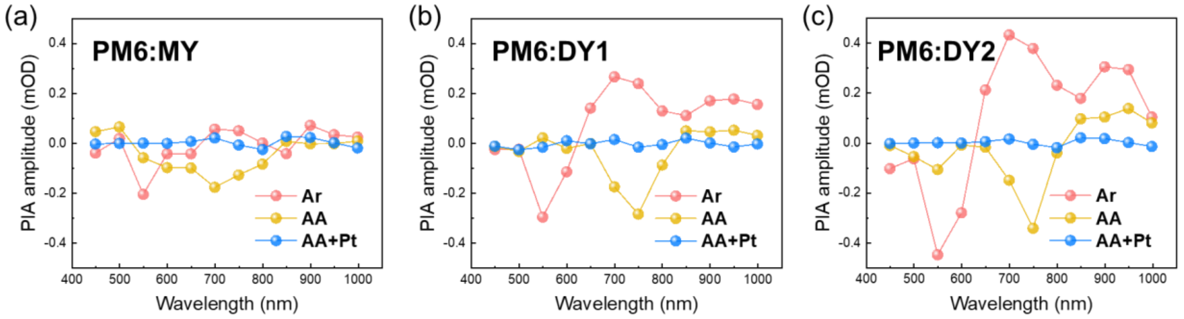

Figure S38. PIA spectra of (a) PM6:MY, (b) PM6:DY1, and (c) PM6:DY2 NPs under 530 nm LED excitation in the absence of both AA and Pt (red), in the presence of AA only (yellow), and in the presence of both AA and Pt (blue). All measurements were performed under Ar-purged conditions.

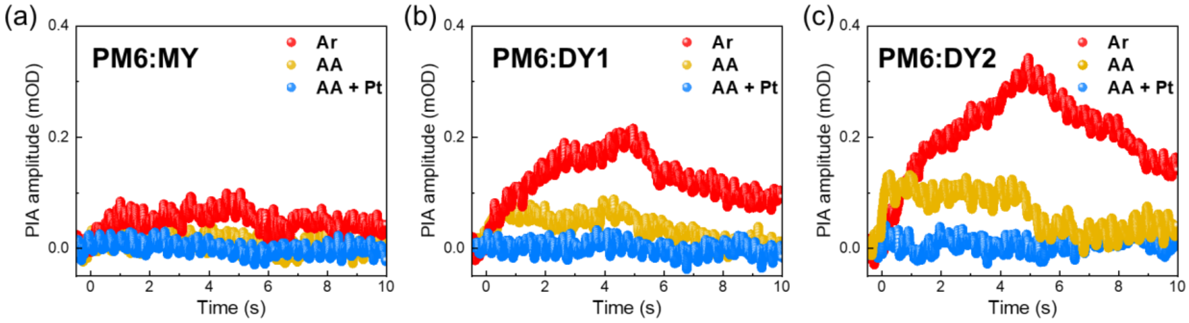

Figure S39. PIA kinetics of (a) PM6:MY, (b) PM6:DY1, and (c) PM6:DY2 NPs measured at 900 nm under 530 nm LED excitation in the absence of both AA and Pt (red), in the presence of AA only (yellow), and in the presence of both AA and Pt (blue).

References

[1] a)J. Yuan, Y. Zhang, L. Zhou, G. Zhang, H.-L. Yip, T.-K. Lau, X. Lu, C. Zhu, H. Peng, P. A. Johnson, M. Leclerc, Y. Cao, J. Ulanski, Y. Li, Y. Zou, *Joule* **2019**, 3, 1140; b)S. R. Adusumalli, D. G. Rawale, U. Singh, P. Tripathi, R. Paul, N. Kalra, R. K. Mishra, S. Shukla, V. Rai, *J. Am. Chem. Soc.* **2018**, 140, 15114; c)G.-U. Kim, C. Sun, J. S. Park, H. G. Lee, D. Lee, J.-W. Lee, H. J. Kim, S. Cho, Y.-H. Kim, S.-K. Kwon, B. J. Kim, *Adv. Funct. Mater.* **2021**, 31, 2100870; d)Y. Li, J. L. Song, Y. C. Dong, H. Jin, J. M. Xin, S. J. Wang, Y. H. Ca, L. Jiang, W. Ma, Z. Tang, Y. M. Sun, *Adv. Mater.* **2022**, 34, 2110155; e)X. Zhao, Y. Zhao, Q. Ge, K. Butrouna, Y. Diao, K. R. Graham, J. Mei, *Macromolecules* **2016**, 49, 2601; f)C. Sun, J.-W. Lee, C. Lee, D. Lee, S. Cho, S.-K. Kwon, B. J. Kim, Y.-H. Kim, *Joule* **2023**, 7, 416.

[2] L. Wang, R. Fernández-Terán, L. Zhang, D. L. A. Fernandes, L. Tian, H. Chen, H. Tian, *Angew. Chem. Int. Ed.* **2016**, 55, 12306.

[3] P. B. Pati, G. Damas, L. Tian, D. L. A. Fernandes, L. Zhang, I. B. Pehlivan, T. Edvinsson, C. M. Araujo, H. Tian, *Energy Environ. Sci.* **2017**, 10, 1372.

[4] C. B. Meier, R. S. Sprick, A. Monti, P. Guiglion, J.-S. M. Lee, M. A. Zwijnenburg, A. I. Cooper, *Polymer* **2017**, 126, 283.

[5] J. Kosco, I. McCulloch, *ACS Energy Lett.* **2018**, 3, 2846.

[6] C. Cheng, X. Wang, Y. Lin, L. He, J.-X. Jiang, Y. Xu, F. Wang, *Polym. Chem.* **2018**, 9, 4468.

[7] P.-J. Tseng, C.-L. Chang, Y.-H. Chan, L.-Y. Ting, P.-Y. Chen, C.-H. Liao, M.-L. Tsai, H.-H. Chou, *ACS Catal.* **2018**, 8, 7766.

[8] A. Liu, C.-W. Tai, K. Holá, H. Tian, *J. Mater. Chem. A* **2019**, 7, 4797.

[9] P. Zhao, L. Wang, Y. Wu, T. Yang, Y. Ding, H. G. Yang, A. Hu, *Macromolecules* **2019**, 52, 4376.

[10] Z. Wang, N. Mao, Y. Zhao, T. Yang, F. Wang, J.-X. Jiang, *Polym. Bull.* **2019**, 76, 3195.

[11] Z.-A. Lan, W. Ren, X. Chen, Y. Zhang, X. Wang, *Appl. Catal. B Environ.* **2019**, 245, 596.

[12] Z. Hu, Z. Wang, X. Zhang, H. Tang, X. Liu, F. Huang, Y. Cao, *iScience* **2019**, 13, 33.

[13] G. Shu, Y. Li, Z. Wang, J.-X. Jiang, F. Wang, *Appl. Catal. B Environ.* **2020**, 261, 118230.

[14] C.-L. Chang, W.-C. Lin, C.-Y. Jia, L.-Y. Ting, J. Jayakumar, M. H. Elsayed, Y.-Q. Yang, Y.-H. Chan, W.-S. Wang, C.-Y. Lu, P.-Y. Chen, H.-H. Chou, *Appl. Catal. B Environ.* **2020**, 268, 118436.

[15] H. Yang, X. Li, R. S. Sprick, A. I. Cooper, *Chem. Commun.* **2020**, 56, 6790.

[16] J. Kosco, M. Bidwell, H. Cha, T. Martin, C. T. Howells, M. Sachs, D. H. Anjum, S. Gonzalez Lopez, L. Zou, A. Wadsworth, W. Zhang, L. Zhang, J. Tellam, R. Sougrat, F. Laquai, D. M. DeLongchamp, J. R. Durrant, I. McCulloch, *Nat. Mater.* **2020**, 19, 559.

[17] M. H. Elsayed, J. Jayakumar, M. Abdellah, T. H. Mansoure, K. Zheng, A. M. Elewa, C.-L. Chang, L.-Y. Ting, W.-C. Lin, H.-h. Yu, W.-H. Wang, C.-C. Chung, H.-H. Chou, *Appl. Catal. B Environ.* **2021**, 283, 119659.

[18] J. Yang, H. Su, Y. Dong, Y. Fu, X. Guo, H. Sun, S. Yin, *New J. Chem.* **2021**, 45, 1423.

[19] M. H. Elsayed, M. Abdellah, Y.-H. Hung, J. Jayakumar, L.-Y. Ting, A. M. Elewa, C.-L. Chang, W.-C. Lin, K.-L. Wang, M. Abdel-Hafiez, H.-W. Hung, M. Horie, H.-H. Chou, *ACS Appl. Mater. Interfaces* **2021**, 13, 56554.
[20] Y. Hu, Y. Liu, J. Wu, Y. Li, J. Jiang, F. Wang, *ACS Appl. Mater. Interfaces* **2021**, 13, 42753.
[21] M. Yu, W. Zhang, Z. Guo, Y. Wu, W. Zhu, *Angew. Chem. Int. Ed.* **2021**, 60, 15590.
[22] A. Liu, L. Gedda, M. Axelsson, M. Pavliuk, K. Edwards, L. Hammarström, H. Tian, *J. Am. Chem. Soc.* **2021**, 143, 2875.
[23] Q. Sheng, Y. Du, Y. Dong, J. Zhao, X. Zhong, Y. Xie, *Appl. Surf. Sci.* **2022**, 603, 154425.
[24] S. An, S. Z. Hassan, J.-W. Jung, H. Cha, C.-H. Cho, D. S. Chung, *Small Methods* **2022**, 6, 2200010.
[25] A. Dolan, J. M. de la Perrelle, T. D. Small, E. R. Milsom, G. F. Metha, X. Pan, M. R. Andersson, D. M. Huang, T. W. Kee, *ACS Appl. Nano Mater.* **2022**, 5, 12154.
[26] J. M. de la Perrelle, A. Dolan, E. R. Milsom, T. D. Small, G. F. Metha, X. Pan, M. R. Andersson, D. M. Huang, T. W. Kee, *J. Phys. Chem. C* **2022**, 126, 14518.
[27]A. M. Elewa, C.-Y. Liao, W.-L. Li, I. M. A. Mekhemer, H.-H. Chou, *Macromolecules* **2023**, 56, 1352.
[28] J. Kosco, S. Gonzalez-Carrero, C. T. Howells, T. Fei, Y. Dong, R. Sougrat, G. T. Harrison, Y. Firdaus, R. Sheelamanthula, B. Purushothaman, F. Moruzzi, W. Xu, L. Zhao, A. Basu, S. De Wolf, T. D. Anthopoulos, J. R. Durrant, I. McCulloch, *Nat. Energy* **2022**, 7, 340.
[29] J. Kosco, S. Gonzalez-Carrero, C. T. Howells, W. Zhang, M. Moser, R. Sheelamanthula, L. Zhao, B. Willner, T. C. Hidalgo, H. Faber, B. Purushothaman, M. Sachs, H. Cha, R. Sougrat, T. D. Anthopoulos, S. Inal, J. R. Durrant, I. McCulloch, *Adv. Mater.* **2022**, 34, 2105007.
[30] Y. Yang, D. Li, P. Wang, X. Zhang, H. Zhang, B. Du, C. Guo, T. Wang, D. Liu, *Polymer* **2022**, 244, 124667.
[31] Z. Zhang, W. Si, B. Wu, W. Wang, Y. Li, W. Ma, Y. Lin, *Angew. Chem. Int. Ed.* **2022**, 61, e202114234.
[32] Y. Zhu, Z. Zhang, W. Si, Q. Sun, G. Cai, Y. Li, Y. Jia, X. Lu, W. Xu, S. Zhang, Y. Lin, *J. Am. Chem. Soc.* **2022**, 144, 12747.
[33] S. An, Z. Wu, H. Jeong, J. Lee, S. Y. Jeong, W. Lee, S. Kim, J. W. Han, J. Lim, H. Cha, H. Y. Woo, D. S. Chung, *Small* **2023**, 19, 2204905.
[34] A. Brnovic, L. Hammarström, H. Tian, *J. Phys. Chem. C* **2023**, 127, 12631.
[35] M. V. Pavliuk, S. Wrede, H. Tian, *Chem. Commun.* **2023**, 59, 5611.
[36] A. Liu, S. Wang, H. Song, Y. Liu, L. Gedda, K. Edwards, L. Hammarström, H. Tian, *Phys. Chem. Chem. Phys.* **2023**, 25, 2935.
[37] Y. Yang, D. Li, J. Cai, H. Wang, C. Guo, S. Wen, W. Li, T. Wang, D. Liu, *Adv. Funct. Mater.* **2023**, 33, 2209643.
[38] X. Liu, Y. Zhao, Y. Ni, F. Shi, X. Guo, C. Li, *Energy Environ. Sci.* **2023**, 16, 4065.
[39]Y. Li, Z. Zhang, T. Li, Y. Liang, W. Si, Y. Lin, *Angew. Chem. Int. Ed.* **2023**, 62, e202307466.
[40] Y. Liang, T. Li, Y. Lee, Z. Zhang, Y. Li, W. Si, Z. Liu, C. Zhang, Y. Qiao, S. Bai, Y. Lin, *Angew. Chem. Int. Ed.* **2023**, 62, e202217989.

[41] X. Yuan, K. Yang, C. Grazon, C. Wang, L. Vallan, J.-D. Isasa, P. M. Resende, F. Li, C. Brochon, H. Remita, G. Hadziioannou, E. Cloutet, J. Li, *Angew. Chem. Int. Ed.* **2024**, 63, e202315333.
[42] M. Axelsson, Z. Xia, S. Wang, M. Cheng, H. Tian, *JACS Au* **2024**, 4, 570.
[43] A. Dolan, X. Pan, M. J. Griffith, A. Sharma, J. M. de la Perrelle, D. Baran, G. F. Metha, D. M. Huang, T. W. Kee, M. R. Andersson, *Adv. Mater.* **2024**, 36, 2309672.
[44] M. H. Elsayed, M. Abdellah, A. Z. Alhakemy, I. M. A. Mekhemer, A. E. A. Aboubakr, B.-H. Chen, A. Sabbah, K.-H. Lin, W.-S. Chiu, S.-J. Lin, C.-Y. Chu, C.-H. Lu, S.-D. Yang, M. G. Mohamed, S.-W. Kuo, C.-H. Hung, L.-C. Chen, K.-H. Chen, H.-H. Chou, *Nat. Commun.* **2024**, 15, 707.
[45] H. Sun, J. Fan, R. Fan, P. Sun, S. Wang, D. Wang, P. Gu, W. Tan, Y. Zhu, *Angew. Chem. Int. Ed.* **2025**, 64, e202503792.
[46] A. Holmes, J. Pan, L. Wang, L. Franco, R. R. Bicudo, B. Albinsson, C. M. Araujo, W. Zhu, D. Wang, T.-Q. Nguyen, J. Zhu, E. Wang, *Adv. Mater.* **2025**, 37, 2507702.
[47] Y. Wang, P. Maity, Y. Jia, B. Liu, L. Zhao, Y. Li, W. Li, Z. Fei, M. Heeney, S. P. Nunes, W.-L. Li, O. F. Mohammed, H. Zhang, *Sci. Adv.* **2025**, 11, eaea4191.
[48] Z. Zhang, C. Xu, Q. Sun, Y. Zhu, W. Yan, G. Cai, Y. Li, W. Si, X. Lu, W. Xu, Y. Yang, Y. Lin, *Angew. Chem. Int. Ed.* **2024**, 63, e202402343.
[49] B. Cai, A. Brnovic, M. V. Pavliuk, L. Hammarström, L. Kloo, S. A. Barnett, H. Tian, *Nat. Chem.* **2026**, DOI: 10.1038/s41557.
